# A Curcumin‐Derived HSP70 Inhibitor Disrupts Lysosomal Function to Suppress Triple‐Negative Breast Cancer Progression

**DOI:** 10.1111/cpr.70222

**Published:** 2026-04-29

**Authors:** Zijian Li, Wanxia Wang, Yuxin Zhou, Tao Zeng, Jian Liu, Yingjie Qing, Xuan Han

**Affiliations:** ^1^ School of Integrative Medicine Nanjing University of Chinese Medicine Nanjing China; ^2^ State Key Laboratory of Pharmaceutical Biotechnology College of Life Sciences, Nanjing University Nanjing China; ^3^ State Key Laboratory of Technologies for Chinese Medicine Pharmaceutical Process Control and Intelligent Manufacture Nanjing University of Chinese Medicine Nanjing China

**Keywords:** autophagy‐lysosome, chemotherapy, curcumin derivatives, HSP70, metastatic triple‐negative breast cancer

## Abstract

Structural modification of curcumin yielded a novel series of 1,4‐pentadien‐3‐one oxime ether derivatives, among which compound M4 exhibited exceptional antitumor activity against triple‐negative breast cancer (TNBC). M4 demonstrated selective cytotoxicity against TNBC cells, inducing apoptosis and significantly reducing tumour progression and pulmonary metastasis in a 4 T1 orthotopic mouse model at doses lower than paclitaxel. Mechanistic investigations revealed that M4 directly targets heat shock protein 70 (HSP70) by binding to its ATPase domain, triggering lysosomal dysfunction characterized by pH neutralization, reduced acid sphingomyelinase activity, lipid accumulation, and cathepsin leakage. These alterations led to disruption of lysosomal function leading to impaired autophagic degradation, as evidenced by the accumulation of autophagosomes and increased levels of LC3‐II/p62. Knockdown of the HSP70 gene abolished M4‐induced lysosomal damage and its anti‐TNBC effects, confirming HSP70 as the functional target of M4. Furthermore, inhibition of HSP70 suppressed TNBC metastasis by regulating autophagy‐mediated epithelial‐mesenchymal transition and stemness. M4 demonstrated selective cytotoxicity against TNBC cells, induced apoptosis, and significantly inhibited tumour progression and pulmonary metastasis in a 4 T1 orthotopic mouse model at doses lower than those of paclitaxel. The combination of M4 and paclitaxel showed synergistic anti‐TNBC efficacy both in vitro and in vivo, effectively counteracting chemotherapy‐induced HSP70 upregulation and autophagic activation. The study not only identifies M4 as a promising therapeutic candidate but also validates HSP70‐targeted therapy as an effective combinatorial strategy with conventional chemotherapy for the treatment of TNBC.

## Graphical abstract

Novel curcumin‐derived M4 binds HSP70's ATPase domain, disrupting HSP70‐mediated lysosomes‐autophagy pathway to inhibit TNBC growth and metastasis, and synergizes with paclitaxel for potent combination therapy.

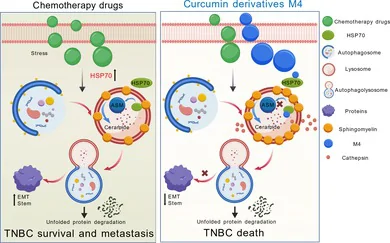

## 1 Introduction

Triple‐negative breast cancer (TNBC) is an aggressive and biologically diverse subtype of breast cancer characterized by the absence of oestrogen receptor, progesterone receptor, and human epidermal growth factor receptor 2 expression. This lack of key hormone and growth factor receptors not only contributes to its highly malignant behaviour but also severely restricts the availability of targeted treatment options, rendering TNBC significantly challenging to manage and frequently associated with unfavourable clinical outcomes [1, 2]. Patients with TNBC frequently experience disease recurrence and distant metastasis, both of which are significant contributors to reduced overall survival rates. These clinical challenges highlight an urgent need for developing novel therapeutic agents that can effectively target this aggressive cancer subtype [3, 4, 5].

The primary treatment option for TNBC is chemotherapy [6]. Autophagy, a cellular process that degrades damaged organelles and misfolded proteins, plays a key role in the response of TNBC to chemotherapy [7]. Chemotherapy often induces autophagy in TNBC cells, allowing them to resist treatment and develop drug resistance. Research indicates that combining chemotherapy with autophagy inhibitors improves treatment efficacy by blocking this survival mechanism, making cancer cells more sensitive to treatment and reducing the risk of metastasis [8, 9]. Using autophagy inhibitors alongside chemotherapy is a promising advance in TNBC management, highlighting the need for further research into this strategy.

Heat shock protein 70 (HSP70) is a highly conserved molecular chaperone involved in cellular stress responses and protein homeostasis. HSP70 is essential for protecting cells against stress‐induced damage and exerts significant regulatory effects on autophagy [10, 11, 12]. The mechanisms through which HSP70 influences autophagy are multifaceted and context‐dependent. On the one hand, HSP70 can facilitate autophagosome maturation, accelerating the clearance of damaged organelles and misfolded proteins [10]. On the other hand, HSP70 is frequently upregulated in TNBC, where it increases autophagic activity to promote cancer cell survival under conditions of therapeutic stress, including chemotherapy or radiation [13, 14]. This adaptive survival mechanism underscores the potential of targeting HSP70 to disrupt cancer cell resilience. Inhibiting HSP70 has emerged as a promising strategy for interfering with this pro‐survival autophagic pathway, leading to increased cancer cell death. Recent advancements in drug discovery have led to the development of novel HSP70 inhibitors, including small‐molecule compounds such as VER‐155008 and JG‐98, which have demonstrated significant efficacy in preclinical studies by selectively impairing HSP70 function and inducing apoptotic cell death in cancer cells [15, 16, 17, 18]. Furthermore, combining HSP70 inhibition with conventional chemotherapy or immunotherapy represents a synergistic approach that may improve treatment outcomes in TNBC [19, 20, 21]. These emerging therapeutic strategies highlight the potential of HSP70 as a viable and effective molecular target for TNBC treatment.

Curcumin is a polyphenolic compound derived from turmeric, exhibiting significant anti‐breast cancer properties, particularly through its ability to regulate autophagy in cancer cells [21, 22, 23, 24]. Recent studies have highlighted its potential as an adjuvant therapy when combined with chemotherapy [25], it can enhance the sensitivity of drug‐resistant tumour cells and modulate survival pathways, thereby improving the efficacy of conventional chemotherapeutic drugs [26]. However, the clinical application of curcumin is limited by its poor pharmacokinetic properties: low water solubility leads to low bioavailability, and rapid metabolic degradation results in insufficient drug accumulation at the tumour site [27, 28, 29]. Additionally, achieving therapeutic concentrations often requires high‐dose administration, potentially increasing toxicity risks and narrowing the therapeutic window. To overcome these challenges, researchers are developing curcumin derivatives with optimized pharmacological properties, including improved efficacy, increased solubility, prolonged half‐life, and targeted delivery systems, to maximize efficacy while minimizing systemic toxicity [30, 31].

In this study, a novel series of 1,4‐pentadien‐3‐one oxime ether derivatives (designated as compounds M) was designed and synthesized by introducing a quinazoline moiety to the core scaffold of curcumin. Focusing on breast cancer, particularly TNBC, compound M4 was identified as a lead molecule with selective cytotoxicity against TNBC cells. M4 inhibits autophagic flux by disrupting lysosomal function. Mechanistic studies revealed that M4 exerts its anti‐cancer effects by binding to HSP70, thereby disrupting lysosomal integrity and function, and thus impeding autophagic degradation processes. Additionally, when combined with conventional chemotherapeutic agents, M4 synergistically enhanced the suppression of breast cancer cell growth. These findings highlight M4 as a promising new compound for targeting HSP70 and offer a potential therapeutic strategy for treating breast cancer.

## 2 Materials and Methods

### 2.1 Cell Lines and Culture

The human breast cancer cell line MDA‐MB‐231 and mouse breast cancer cell line 4 T1 were obtained from the American Type Culture Collection (Manassas, VA, USA). All cells were cultured in Dulbecco's modified Eagle medium supplemented with 10% heat‐inactivated fetal bovine serum (FBS) and 100 μg/mL penicillin/streptomycin at 37°C in a humidified incubator with 5% CO_2_. Cells were passaged three times before conducting other experiments.

### 2.2 Plasmid Construction and Establishment of Stable Cell Lines

To elucidate the biological role of HSP70, we developed overexpression and knockdown constructs. The coding sequence of human HSP70 was cloned into a pCMV‐based vector to generate an overexpression plasmid. For knockdown experiments, shRNAs targeting HSP70 were designed and inserted into the pLKO.1‐puro lentiviral vector, with a scrambled shRNA construct serving as the control. All plasmid constructs were confirmed using DNA sequencing. Lentiviral particles were produced by co‐transfecting the respective plasmids with packaging vectors into HEK‐293 T cells. Supernatants containing viral particles were collected, concentrated, and used to transduce target cells. Stable polyclonal populations were selected under puromycin treatment, and successful modulation of HSP70 expression, either overexpression or knockdown, was confirmed using Western blotting (WB).

### 2.3 Synthesis and Ex Vivo Imaging

Quinoxaline‐6,7‐diol was employed as the starting material and treated with an excess of thionyl chloride (SOCl_2_) in the presence of a catalytic amount of N, N‐dimethylformamide (DMF). The mixture was heated under reflux for 3–5 h under a nitrogen atmosphere. Upon completion, excess SOCl_2_ was removed by distillation under reduced pressure. The residue was poured into ice water, and the resulting solid was collected by filtration, washed with cold water, and dried to afford intermediate A (2,6,7‐trichloroquinoxaline) as an off‐white solid, which was used directly in the next step.

For the synthesis of the target compounds M1–M26, a solution of intermediate A (1.0 mmol, 1.0 equiv) and the corresponding substituted benzaldehyde derivative (intermediate 1, 1.2 mmol, 1.2 equiv) in anhydrous acetonitrile (30 mL) was added potassium carbonate (K_2_CO_3_, 2.0 mmol, 2.0 equiv). The mixture was heated to reflux (82°C–85°C) under a nitrogen atmosphere, and the reaction progress was monitored by thin‐layer chromatography (TLC) (eluent: petroleum ether/ethyl acetate, ratio adjusted based on polarity). Typically, after 4–6 h, the reaction mixture was cooled to room temperature and filtered to remove inorganic salts. The filtrate was concentrated under reduced pressure to obtain the crude product, which was purified by column chromatography on silica gel (200–300 mesh, eluent: petroleum ether/ethyl acetate, gradient elution) to yield the final target compounds M1–M26.

Structural confirmation was performed using a Bruker Avance NEO 400 MHz NMR spectrometer with deuterated dimethyl sulfoxide as the solvent and tetramethylsilane as the internal standard. Structural elucidation was completed through analysis of proton nuclear magnetic resonance (^1^H NMR), carbon‐13 NMR (^13^C NMR), and two‐dimensional NMR spectra (COSY, HSQC, and HMBC). All spectral data were analysed using MestReNova software, and the results confirmed the predicted chemical structures of M4 and M5.

### 2.4 WB Assay

The treated cells or tissue samples were lysed on ice using radio‐immunoprecipitation assay (RIPA) lysis buffer supplemented with 1% protease and phosphatase inhibitors. The lysates were centrifuged, and the supernatants were collected. Protein concentration was determined using a bicinchoninic acid protein assay kit. Equal amounts of protein (20–30 μg) were separated using 10%–12% sodium dodecyl sulfate‐polyacrylamide gel electrophoresis and transferred onto polyvinylidene fluoride membranes. The membranes were blocked with 5% bovine serum albumin for 1 h at room temperature, followed by overnight incubation with the primary antibodies at 4°C. After washing with TBST, the membranes were incubated with HRP‐conjugated secondary antibodies for 1–2 h at room temperature. Protein bands were visualized using a chemiluminescent substrate system and imaged.

### 2.5 Immunofluorescence Assay

The cells treated with the specified concentrations of compound M4 were washed with cold phosphate‐buffered saline (PBS), fixed with 4% paraformaldehyde for 15 min at room temperature, and permeabilized with 0.1% Triton X‐100 for 10 min. Subsequently, the cells were blocked with 10% goat serum for 1 h at room temperature to prevent non‐specific binding. The blocked cells were then incubated overnight at 4°C with primary antibodies against Galectin‐3 (for autophagic flux) and CTSB/cathepsin B (for lysosomes). After washing with PBS, the cells were incubated with Alexa Fluor 488‐conjugated secondary antibodies for 1 h at room temperature in the dark. Finally, the cell nuclei were counterstained with DAPI solution, and the images were captured using a laser scanning confocal microscope. The number of Gal3 or CTSB puncta per cell was quantified using ImageJ software.

### 2.6 CTC Cluster Mode

Human breast cancer cells MDA‐MB‐231 were detached using ethylenediaminetetraacetic acid buffer (0.05 mmol/L) to prepare single‐cell suspensions, which were then seeded in poly‐HEMA‐precoated 96‐well ultra‐low attachment plates for suspension culture. The experiment included two groups: negative control (NC) and HSP70 knockdown (shHSP70). Cell culture was continuously monitored using the IncuCyte live‐cell imaging system with automated image acquisition every 2 h. Dynamic changes in cluster size were quantitatively analysed using IncuCyte ZOOM software.

### 2.7 Microscale Thermophoresis (MST)

MST (Microscale thermophoresis) assay was performed using a Monolith NT.115 instrument. HSP70 was fluorescently labelled according to the manufacturer's protocol. M4 was serially diluted to the required concentrations (from 5nM to 100 μM) and incubated with a fixed concentration (200 nM) of the labelled protein for 15 min in the running buffer. The samples were then loaded into standard capillaries, and measurements were performed using 80% LED and 80% MST power. The dissociation constant (Kd) was calculated from duplicate measurements using the mass action equation integrated in the instrument's software.

### 2.8 DARTS Experiment

Total proteins were extracted from the cultured cells using RIPA lysis buffer supplemented with protease inhibitors. Following centrifugation and quantification, equal amounts of protein lysates were incubated with either M4 or dimethyl sulfoxide control at room temperature for 1 h. Limited proteolysis was performed by adding pronase at protein‐to‐enzyme ratios ranging from 100:1 to 200:1 and incubating at room temperature for 30 min. The reaction was terminated by adding protease inhibitors and placing the samples on ice. Denatured samples were analysed via WB using an HSP70‐specific antibody to measure stability changes across treatment groups. Reverse virtual screening (RVS) was performed using the PharmMapper web (https://www.lilab‐ecust.cn/pharmmapper/index.html) to predict potential human protein targets of compound M4. PharmMapper identifies candidate targets by aligning the chemical features of the query molecule against an internal pharmacophore database derived from druggable protein structures. The targets were ranked based on the pharmacophore fit score, and the top 261 proteins with normalized fit scores ≥ 4.0 were selected for downstream intersection analysis.

### 2.9 Molecular Docking

The molecular interaction between HSP70 protein and M4 small molecule was investigated using molecular docking. The three‐dimensional structure of HSP70 was obtained from the Protein Data Bank (PDB ID: 5TKY). The M4 molecule was constructed and energetically minimized using Chem3D. Semi‐flexible docking was performed using AutoDock Vina with the grid box centered on the HSP70 active site. An exhaustiveness value of 8 was applied, and nine binding poses were generated. The most favourable binding conformation was selected based on calculated binding free energy, and key intermolecular interactions between HSP70 and M4, including hydrogen bonds and hydrophobic contacts, were analysed using PyMOL.

### 2.10 Transwell Chamber Assay

A Transwell chamber system was used to evaluate the migratory abilities of cells in vitro. The lower chamber was filled with complete medium containing 10%–15% FBS as a chemoattractant. Following HSP70 overexpression and subsequent treatment with autophagy inhibitors, MDA‐MB‐231 cells were harvested and resuspended in serum‐free medium. The cells were then seeded in the upper chamber at a density of 1 × 10^5^ cells per insert. After incubation for 24–48 h at 37°C with 5% CO_2_, non‐migrated cells on the upper surface of the membrane were removed using a cotton swab. The cells that migrated to the lower surface were fixed using 4% paraformaldehyde and stained with 0.1% crystal violet. Images of multiple random fields per membrane were captured using an inverted microscope, and the number of migrated cells was quantified using the ImageJ software. All experiments were performed in triplicate and independently repeated three times.

### 2.11 Animal Studies

All animal experiments were conducted according to the National Institutes of Health Guide for the Care and Use of Laboratory Animals and were approved by the Institutional Animal Care and Use Committee of Bengbu Medical University (approval No [2024] No. 515). Female BALB/c mice (4 weeks old) were housed under specific pathogen‐free conditions with a 12 h light/dark cycle and allowed to acclimatize for 1 week. To establish an orthotopic breast cancer metastasis model, exponentially growing 4 T1 cells were collected. A suspension containing 1 × 10^6^ cells in 100 μL was orthotopically inoculated into the fourth mammary fat pad of each mouse. When primary tumours reached approximately 80–100 mm^3^, tumour‐bearing mice were randomly allocated to the various experimental groups based on their tumour volume and body weight to ensure comparable baselines without statistical differences at the start of treatment (*n* = 6 per group): Treatment regimens were as follows: Monotherapy groups: (i) Vehicle control; (ii) M4 (5 mg/kg); (iii) M4 (10 mg/kg); (iv) Paclitaxel (PTX, 13.5 mg/kg). Mechanistic interaction groups: (i) Vector‐Control; (ii) Vector‐M4; (iii) shHSP70‐Control; (iv) shHSP70‐M4. Mice were intratumorally injected with AAV carrying control or HSP70‐targeting shRNA 1 week prior to M4 treatment. Combination therapy groups: (i) Vehicle control; (ii) PTX (13.5 mg/kg); (iii) PTX + AAV‐shHSP70; (iv) PTX + M4 (5 mg/kg). M4 was administered via intraperitoneal injection once per week. PTX was administered via tail vein injection once per week. Tumour volumes and body weights were measured every other day throughout the study period. And tumour volume (V) was calculated according to the formula: V = (L × W^2^)/2. All mice were euthanized and dissected in the 7th week of the experiment. The lungs were fixed in Bouin's solution for 12 h and washed with 70% ethanol to determine lung metastasis. The livers were formalin‐fixed, and paraffin‐embedded sections were prepared for histological examination using haematoxylin and eosin (H&E) staining. During the study, the researchers responsible for tumour volume measurements and terminal histological evaluations (e.g., metastasis counting) were blinded to the specific group assignments of the mice until all data analyses were completed.

### 2.12 Statistical Analysis

Statistical analysis was performed using GraphPad Prism software (V.8.0). All results are expressed as the mean ± SEM of three independent experiments. One‐way analysis of variance followed by Dunnett's post hoc test was used to evaluate the differences when there were more than two groups. The Student's *t*‐test was used to evaluate the significant difference between the two groups. A statistical significance was set at *p* < 0.05.

## 3 Results

### 3.1 Curcumin‐Derived Compound M4 Exhibits Potent Anti‐Cancer Properties Against TNBC Similar to Paclitaxel

**FIGURE 1:**
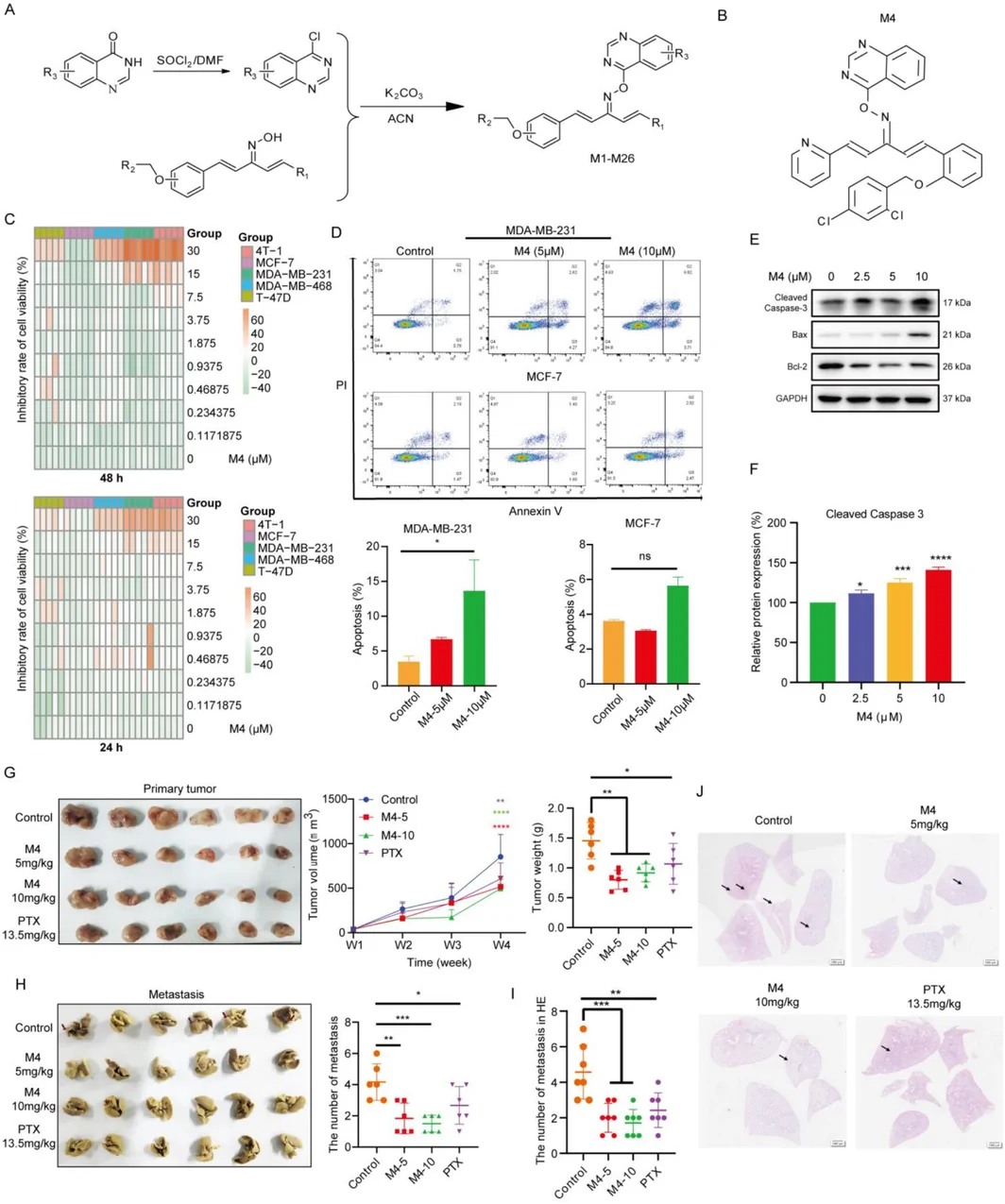
The curcumin‐derived compound M4 exhibits potent anti‐cancer properties against TNBC similar to paclitaxel. (A) Chemical synthesis routes for the M series of curcumin derivatives. (B) Chemical structure of M4 compound. (C) MTT assay was used to evaluate the proliferation‐inhibiting effects of compound M4 on TNBC and non‐TNBC cell lines. TNBC: MDA‐MB‐231/MDA‐MB‐468/4 T1 cell line. Non‐TNBC: MCF‐7/T‐47D cell line. Data are presented as the mean ± SD (*n* = 3 independent experiments). (D) Effects of M4 compound on apoptosis levels in MDA‐MB‐231 and MCF7 cell lines were detected using flow cytometry. (E) WB assay for evaluating the effects of compound M4 on apoptosis‐related proteins in the MDA‐MB‐231 cell line. (F) Quantitative diagram depicting the effects of M4 compound on apoptosis‐related proteins. (G) Anti‐proliferative effects of different doses of M4 compound on primary 4 T1 in situ breast cancer tumours. PTX served as the positive control for chemotherapy. Tumour volume and mass were quantified at the completion of animal experiments (*n* = 6 mice per group). Data are presented as the mean ± SEM. (H) Effects of different doses of M4 compound on distant lung metastasis in 4 T1 in situ breast cancer. At the experimental endpoint, lung metastatic lesions were quantified using macroscopic observation (*n* = 6 mice per group). Data are presented as the mean ± SEM. (I) H&E staining of pulmonary metastases and metastatic lesions. (J) H&E staining of pulmonary metastases. Bar, SD. *p < 0.05, **p < 0.01, ***p < 0.001, ****p < 0.0001 versus the untreated control. ns = no significance. Statistical significance was determined by one‐way ANOVA (C, F—H) or two‐way repeated measures ANOVA (G, tumour growth curves).

Previous studies have elucidated the significant antitumor potential of N‐series curcumin derivatives, particularly N17, against breast cancer [32]. In continuation of this work, we structurally modified the curcumin scaffold by incorporating a quinazoline moiety, designing and synthesizing a novel class of 1,4‐pentadien‐3‐one oxime ether derivatives (designated as M1–M26) to evaluate their cytotoxic effects on breast cancer cells and identify more potent analogs. The synthetic route is depicted in Figure 1A. A mixture of intermediate D (1.07 mmol), intermediate F (1.28 mmol), and potassium carbonate (2.14 mmol) in acetonitrile (30 mL) was stirred at room temperature for 30 min and heated under reflux. Reaction progress was monitored using TLC (petroleum ether/ethyl acetate, 1:1). The reaction was terminated after 3–5 h, and the mixture was filtered and concentrated under reduced pressure. The crude product was purified using column chromatography to obtain compounds M1–M26.

Initially, we used ^1^H NMR, ^13^C NMR, and related spectroscopic techniques to confirm the chemical structures of M4 and M5 (Figure 1B, Figure S1). The structural characterization of compound M4 was accomplished through 1H NMR, 13C NMR, and HRMS: Yellow solid, yield: 68%. m. p. 123°C–124°C; 1 H NMR (500 MHz, CDCl3) δ: 8.73 (s, 1H, Cl—Ar—CH=), 8.14 (d, *J* = 16.0 Hz, 1H, Qu—3—H), 8.10–8.06 (m, 1H, Ar—CH=), 7.81–7.76 (m, 2H, Qu—5,6—H), 7.74–7.71 (m, 3H, Qu—7,8—H, Cl—Ar‐3—H), 7.69–7.62 (m, 2H, Ar‐3,5—H), 7.45 (dd, *J* = 7.5, 1.8 Hz, 1H, Cl—Ar‐6—H), 7.38–7.36 (m, 2H, Cl—Ar—4,5—H), 7.32 (ddd, *J* = 8.6, 5.3, 3.5 Hz, 2H, Ar—C=CH, Cl—Ar—C=CH), 7.12 (d, *J* = 16.0 Hz, 1H, Ar—2—H), 7.07–7.02 (m, 1H, Ar—2—H). 13 C NMR (125 MHz, CDCl3) δ: 188.80, 156.53, 154.69, 142.90, 139.90, 139.26, 139.13, 135.50, 133.15, 131.99, 131.32, 130.70, 130.39, 129.99, 129.10, 128.25, 127.88, 127.82, 127.78, 127.24, 124.74, 122.00. HRMS calculated for C25H18ClN2O2 [M + H] + 413.1043, found 413.1051. Specifically, the 1H NMR, 13C NMR, and HRMS spectra of N17 discussed below: Once their structures were elucidated, N‐series compounds (Figure S1) were used as positive controls to systematically assess the effects of M4 and M5 on the proliferation inhibition of breast cancer cell lines. M4 and M5 exhibited significantly stronger anti‐proliferative activity against breast cancer cells than the N‐series compounds (Figure 1C, Figure S1). Among these, M5 demonstrated the most potent inhibitory effect. Conversely, M4 exhibited selective inhibition, specifically targeting breast cancer cell lines, with significantly greater cytotoxicity against TNBC than against non‐TNBC cell lines (Figure 1C). This selectively enhanced killing of TNBC by M4 compared to the broader and stronger anti‐proliferative activity demonstrated by M5 piqued our interest and led us to hypothesize that M4 could be a promising candidate for the treatment of TNBC. To investigate this hypothesis further, we conducted additional experiments to evaluate the selective cytotoxicity of M4 against TNBC cell lines. We investigated the apoptotic effects of M4 on MDA‐MB‐231 and MCF‐7 breast cancer cell lines. M4 induced apoptosis in MDA‐MB‐231 cells in a dose‐dependent manner but exhibited almost no apoptotic effect on MCF‐7 cells (Figure 1D, Figure S1). WB analyses further validated the pro‐apoptotic activity of M4, demonstrating its efficacy in MDA‐MB‐231 and 4 T1 cell lines (Figure 1E,F, Figure S1). Moreover, the M4 compound exhibited relatively mild inhibition of cell proliferation in the normal mammary epithelial cell line MCF‐10A, especially when compared to its effects on breast cancer cells, implying that it may possess a favourable safety profile (Figure S1).

To confirm the potent anti‐TNBC activity of M4, we developed a 4 T1 orthotopic breast cancer model and used paclitaxel as a positive control. The results demonstrated that M4 exhibited a dose‐dependent and highly potent antitumor effect, significantly reducing the volume and weight of orthotopic tumours (Figure 1G). The antitumor efficacy of M4 was comparable to that of paclitaxel, albeit at a lower dose. Moreover, M4 significantly reduced lung metastasis in this model, outperforming paclitaxel (Figure 1H–J). Meanwhile, in vivo processing of M4 demonstrated minimal impact on liver and kidney damage, indicating its safety (Figure S1). These findings imply that M4 represents a promising and potent novel therapeutic agent for the treatment of TNBC.

### 3.2 Potent Anti‐Breast Cancer Effect of M4 Depends on Lysosomal Dysfunction

**FIGURE 2:**
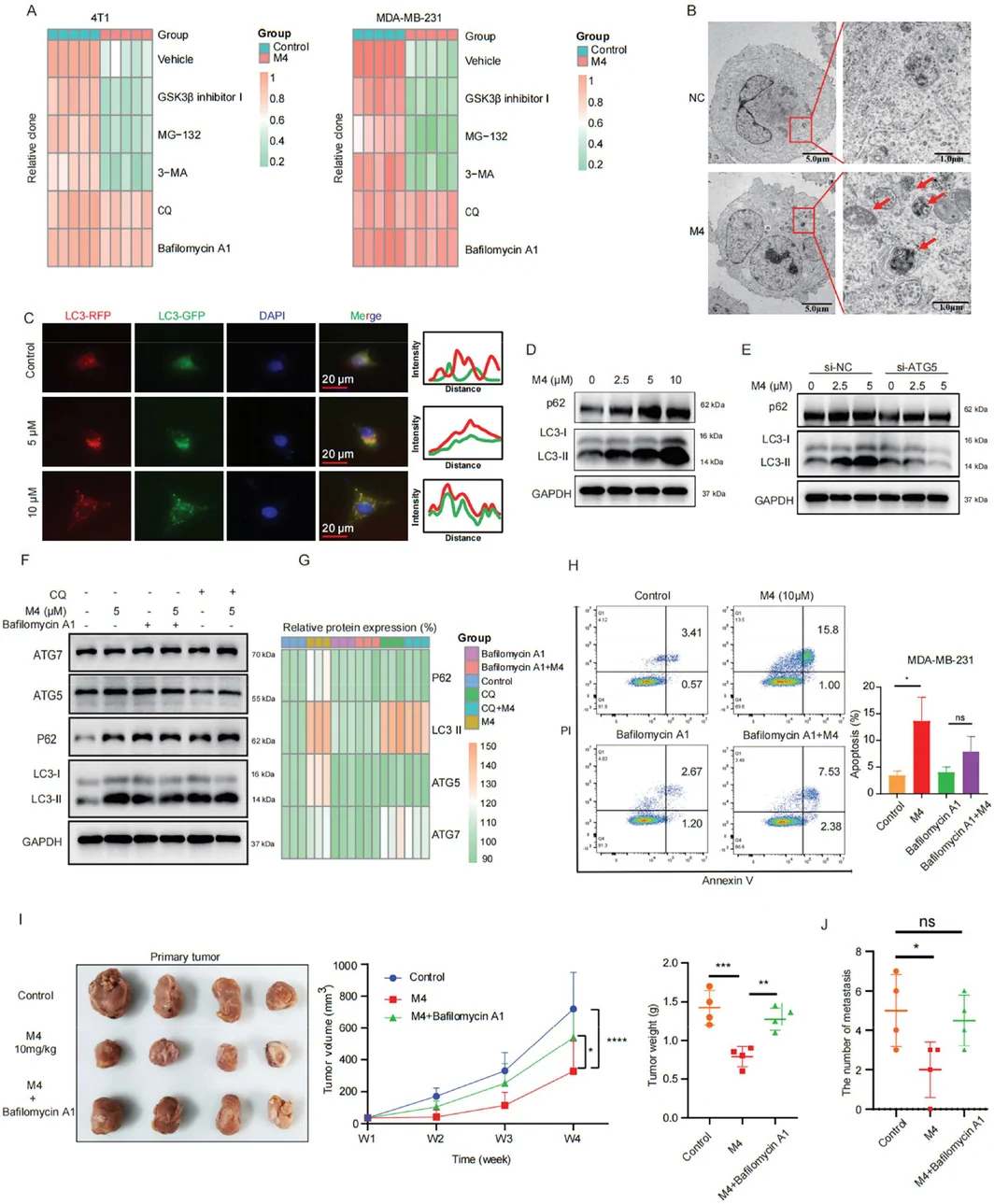
Potent anti‐breast cancer activity of M4 depends on blocking late‐stage autophagy. (A) Pretreatment with various pathway inhibitors was used to investigate the primary pathway responsible for M4's effects on TNBC cell lines (CQ, 20 μM; Baf A1, 100 nM; MG‐132, 10 μM; 3‐MA, 5 mM; GSK3β inhibitor, 10 μM) for 1 h, followed by co‐treatment with M4 for 24 h. Cell viability was assessed by colony formation assay. The results were analysed quantitatively. (B) Transmission electron microscopy analysis of the effects of M4 compound on autophagosomes in the MDA‐MB‐231 cell line. Scale bar: 5.0 and 1.0 μm. (C) Effects of M4 compound treatment on autophagosomes and autophagolysosomes in the MDA‐MB‐231 cell line. GFP fluorescence is quenched in the acidic lysosome, while RFP is stable; therefore, yellow puncta (RFP/GFP colocalization) represent autophagosomes, and red puncta (RFP only) represent autolysosomes. Scale bar: 20 μm. (D) Effects of M4 compound treatment on autophagy‐related proteins LC3B and P62 in the MDA‐MB‐231 cell line. (E) Knockdown of ATG5, a key autophagy gene, was used to determine whether M4 functions through an autophagic pathway in the MDA‐MB‐231 cell line. (F) MDA‐MB‐231 cells were pretreated with DMSO (negative control), autophagy inhibitors chloroquine (CQ, 20 μM) and Bafilomycin A1 (Baf A1, 100 nM) for 1 h, followed by co‐treatment with various concentrations of M4 for 12 h. WB analysis investigating the effects of CQ and bafilomycin on autophagy‐related proteins mediated by the M4 compound. (G) Heatmap illustrating the effects of pretreatment with autophagy inhibitors CQ and bafilomycin on autophagy‐related proteins mediated by the M4 compound. (H) Effect of pretreatment with autophagy inhibitors CQ and bafilomycin on M4 compound‐mediated apoptosis in the MDA‐MB‐231 cell line was measured using flow cytometry. (I) The 4 T1 in situ breast cancer model was used to investigate the impact of bafilomycin treatment on M4‐mediated anti‐TNBC effects. Tumour volume was quantified throughout the experiment, and tumour mass was measured at the end of the study. (J) Quantitative analysis of lung metastases at the experimental endpoint. Data are presented as the mean ± SD (*n* = 6 mice per group). *p < 0.05, **p < 0.01, ***p < 0.001, ****p < 0.0001 versus the untreated control. ns = no significance.

M4 exhibits potent antitumor activity; however, the underlying mechanism remains to be fully elucidated. Based on previous reports that curcumin can disrupt autophagic flux [33, 34], we investigated whether M4 impairs autophagic degradation. In the MDA‐MB‐231 cell line, pretreatment with late‐autophagy inhibitors, unlike GSK3β inhibitors, proteasomal activity inhibitor MG132, and early‐stage autophagy inhibitor 3‐MA (class III PI3K/VPS34 inhibitor) significantly attenuated the anti‐proliferative effect of M4, indicating that its growth‐inhibitory action depends on late‐stage autophagic flux blockade and lysosomal dysfunction (Figure 2A, Figure S1). Furthermore, the inability of 3‐MA to attenuate M4's anti‐tumour effect supports this conclusion.

Further analysis revealed that M4 treatment increased autophagosome accumulation, as demonstrated by electron microscopy and immunofluorescence (Figure 2B,C), without promoting autolysosome formation (Figure 2C). Consistently, WB demonstrated a dose‐dependent increase in LC3‐II and p62 levels, indicating impaired autophagic degradation (Figure 2D,E, Figure S2B). When autophagic flux was blocked by chloroquine (CQ) or bafilomycin A1, the M4‐induced upregulation of ATG5, p62, and LC3‐II was suppressed (Figure 2F, Figures S1 and S2C). Moreover, bafilomycin A1 abolished M4‐induced apoptosis (Figure 2H), confirming that autophagy blockade is required for M4‐induced cell death. These results demonstrate that M4 exerts anti‐TNBC effects by blocking autophagic flux, leading to apoptotic cell death.

In vivo, intratumoral administration of bafilomycin A1 significantly reversed the M4‐induced tumour growth suppression and metastasis inhibition (Figure 2I,J). These findings demonstrate that the potent anti‐TNBC activity of M4 depends on its ability to disrupt autophagic flux.

### 3.3 M4‐Induced Blockade of Autophagic Flux Depends on Lysosomotropic Activity and Its Disruption

**FIGURE 3:**
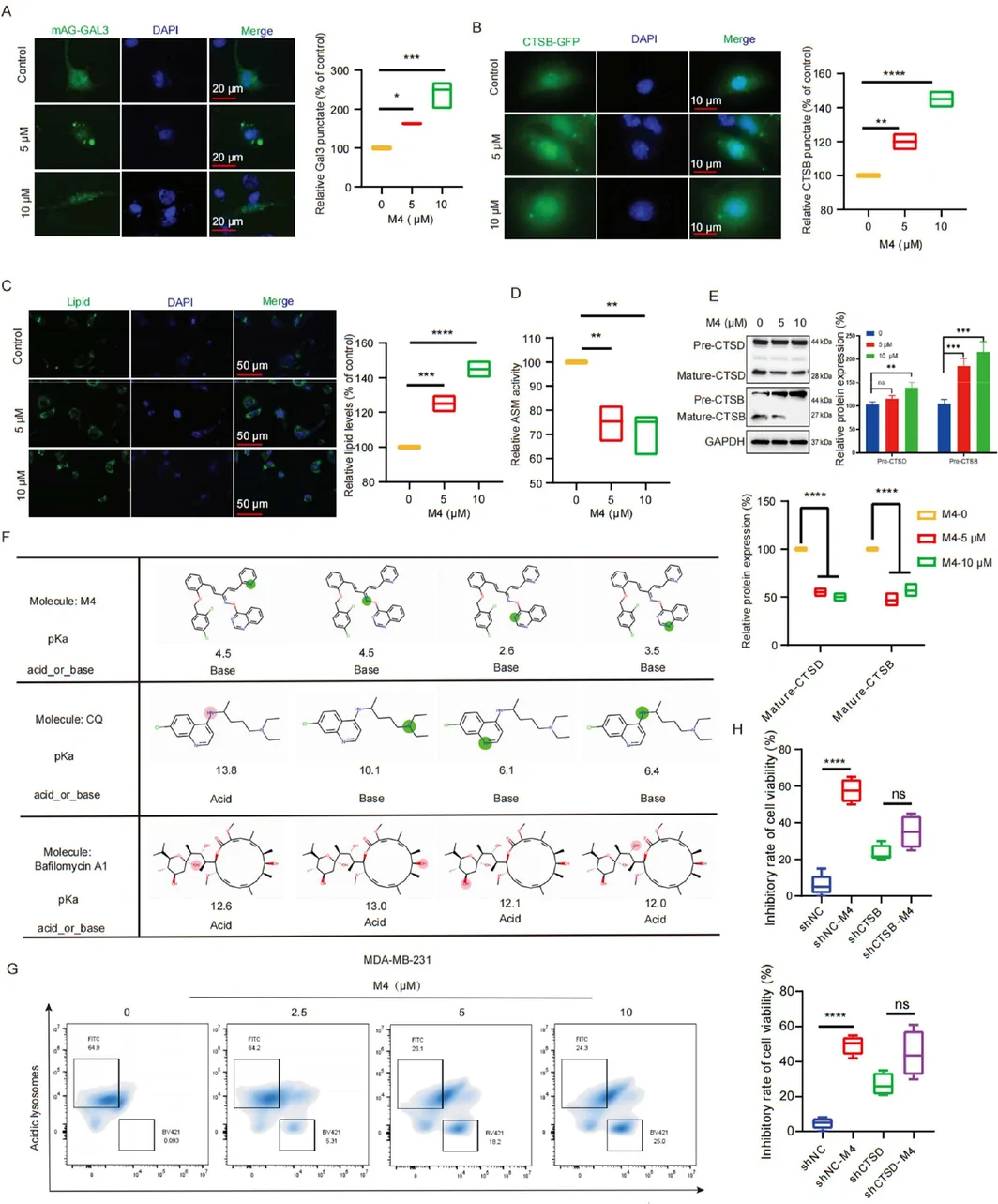
The M4‐mediated blockade of autophagic flux depends on lysosomal activity and its disruption. (A) Immunofluorescence assay depicting and quantifying the promoting effect of M4 treatment on lysosomal damage in the MDA‐MB‐231 cell line. Scale bar: 20 μm. (B) Immunofluorescence detection and quantification of the effect of M4 treatment on CTSB expression in the cytoplasm of MDA‐MB‐231 cells. Scale bar: 10 μm. (C) Immunofluorescence detection and quantification of the effects of M4 treatment on lipid metabolism in the MDA‐MB‐231 cell line. Scale bar: 50 μm. (D) Effects of M4 treatment on ASM activity in the MDA‐MB‐231 cell line. (E) Effects of M4 treatment on the expression levels of CTSB, CTSD, pre‐CTSB, and pre‐CTSD in the MDA‐MB‐231 cell line. (F) The acid–base properties of the structural moieties in M4 compounds were predicted, with CQ and bafilomycin serving as positive controls. (G) MDA‐MB‐231 cells were stained with the dual‐fluorescence probes LysoSensor Yellow/Blue DND‐160 (PDMPO). LysoSensor Yellow were defined as the population with acidic lysosomes, LysoSensor Blue were defined as the population with neutralized lysosomes. The right panel displays the quantitative ratio of neutral to acidic lysosomes. (H) MTT assay was used to investigate the inhibitory effect of M4 on the proliferation of the MDA‐MB‐231 cell line following interference with CTSB and CTSD. Data are presented as the mean ± SD (*n* = 3 independent experiments). *p < 0.05, **p < 0.01, ***p < 0.001, ****p < 0.0001 versus the untreated control. ns = no significance. Statistical significance was determined by one‐way ANOVA.

Based on preliminary findings indicating that M4 induces autophagosome accumulation without facilitating lysosomal fusion, we hypothesized that M4 impairs lysosomal function, leading to autophagic flux disruption. To test this hypothesis, MDA‐MB‐231 cells expressing the lysosomal integrity reporter mAG‐Gal3‐GFP were treated with M4, leading to a significant increase in Gal3‐positive puncta, indicating lysosomal damage (Figure 3A). Lysosomal damage often induces lysosomal membrane permeabilization (LMP) and cathepsin release, a known trigger of lysosome‐dependent cell death (LCD) [35, 36, 37]. To investigate whether M4 promotes LMP, we assessed the subcellular localization of cathepsin B (CTSB) and observed a significant increase in cytoplasmic CTSB‐positive puncta following M4 treatment (Figure 3B). WB analysis revealed elevated levels of cytoplasmic pre‐CTSB (proenzyme form) and immature cathepsin D (pe‐CTSD), indicating that M4 impairs cathepsin maturation and promotes their leakage (Figure 3E). Given the established association between lysosomal membrane stability and lipid metabolism [38, 39, 40], we investigated the effect of M4 on lysosomal membrane lipid homeostasis. M4 treatment induced lipid accumulation and reduced acid sphingomyelinase (ASM) activity (Figure 3C,D), a key regulator of sphingomyelin metabolism [40]. Decreased ASM activity impairs lysosomal membrane integrity and permeability [41]. These results demonstrate that M4 disrupts autophagic flux by impairing lysosomal function through membrane lipid alteration, ASM inhibition, and cathepsin leakage.

Why does M4 induce lysosomal damage? Structural analysis revealed that M4 contains multiple weakly basic groups, a feature similar to known autophagy inhibitors, such as CQ and bafilomycin A1 (Figure 3F). Given the acidic interior of lysosomes, we hypothesized that M4 may accumulate within and neutralize these organelles. Using pH‐sensitive lysosomal probes in MDA‐MB‐231 cells, we discovered that M4 treatment significantly increased the proportion of neutral lysosomes and the neutral‐to‐acidic lysosome ratio (Figure 3G), confirming its ability to disrupt lysosomal acidity. Since lysosomal membrane permeabilization and cathepsin release are key triggers of LCD, we investigated whether lysosomal damage mediates M4‐induced cytotoxicity. Knockdown of CTSB or CTSD in MDA‐MB‐231 cells significantly attenuated the anti‐proliferative effect of M4 (Figure 3H). These results indicate that M4 inhibits cell proliferation primarily by inducing lysosomal alkalization, triggering cathepsin leakage, and promoting apoptosis and autophagic flux blockade.

Furthermore, we investigated the expression levels of cleaved‐caspase 3, CTSD, and LC3B in orthotopic tumour tissues treated with M4 (Figure S5A). The results were consistent with our previous WB data, confirming that M4 induces apoptosis in TNBC cells by disrupting lysosomal function, promoting cathepsin release, and blocking autophagic flux.

### 3.4 Identification of HSP70 as the Direct Binding Target of M4

**FIGURE 4:**
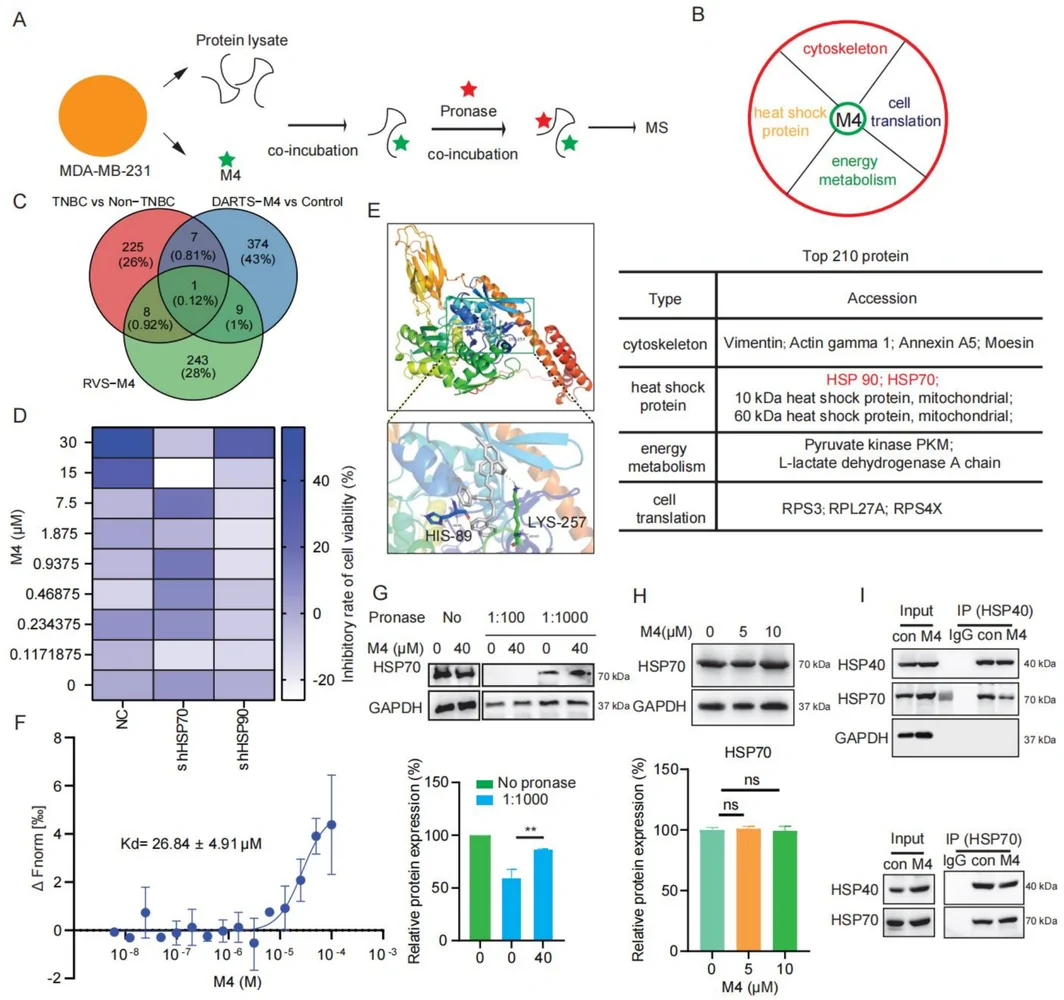
Identification of HSP70 as the direct binding target of M4. (A) DARTS experimental design. (B) Classification of the top 210 proteins identified by M4 processing in the proteomics data. (C) An integrated multi‐omics screening strategy was used to systematically identify the potential protein targets of compound M4 in TNBC. First, the differentially expressed proteins between TNBC and non‐TNBC tumours were extracted from the CPTAC breast cancer cohort. Second, DARTS‐MS was used to profile proteins stabilized upon M4 treatment. Third, RVS was used to predict potential interactors of M4, yielding 261 candidate binding proteins. By overlapping the TNBC‐upregulated proteome, DARTS‐MS hits, and RVS‐predicted targets, we identified the potential direct molecular target of M4 in TNBC. (D) MTT assay was used to investigate the effects of HSP70 and HSP90 interference on the M4‐mediated inhibition of MDA‐MB‐231 cell proliferation. Data are presented as the mean ± SD. (E) Molecular docking was used to investigate the binding between compound M4 and HSP70. (F) MST was used to experimentally confirm the interaction and binding affinity between the M4 compound and HSP70. (G) The DARTS experiment further validated the binding between compound M4 and HSP70. (H) WB analysis to determine the effect of compound M4 on HSP70 protein expression in the MDA‐MB‐231 cell line. (I) Co‐IP assays were used to validate the inhibitory effect of compound M4 on HSP70 function. *p < 0.05, **p < 0.01, ***p < 0.001, ****p < 0.0001 versus the untreated control. ns = no significance. Statistical significance was determined by one‐way ANOVA (D) or unpaired two‐tailed Student's *t*‐test (H, I).

M4 disrupts lysosomal function and inhibits autophagic flux, thereby inducing apoptosis in TNBC cells. Identifying the direct molecular targets underlying these effects is crucial for understanding the mechanism of action. To systematically identify M4‐binding proteins, we used drug affinity responsive target stability (DARTS) coupled with mass spectrometry (DARTS‐MS) (Figure 4A). The results revealed that M4‐interacting proteins were primarily localized to the cytoplasm. Reactome pathway enrichment analysis revealed significant enrichment in caspase activation pathways, consistent with the pro‐apoptotic role of M4 in TNBC cells (Figure S5A–C). To prioritize potential targets, we focused on the top 500 most abundant proteins from DARTS‐MS, which were primarily associated with cytoskeletal organization, heat shock response, energy metabolism, and translation (Figure 4B). To further narrow down TNBC‐relevant targets, we analysed data of patients with TNBC from The Cancer Genome Atlas (TCGA) and identified 241 proteins highly expressed in TNBC (Table S1). The intersection of these two datasets yielded eight overlapping candidates: ASNS, CD44, EEF2, VASP, CDK1, HSP70, TRIM25, and PGM1 (Figure 4C). Among these, HSP70, CD44, EGFR, SRC, and TRIM25 were most strongly associated with autophagy and lysosomal function [42, 43, 44, 45]. Notably, by integrating the TNBC patient CPTAC data, DARTS‐MS, and RVS‐predicted targets, HSP70 was identified as the sole protein present at the intersection of all three datasets (Figure S2G). Given the high abundance of HSPs in the DARTS‐MS dataset, we investigated their potential as direct targets. Genetic inhibition of HSP90 or HSP70 in MDA‐MB‐231 cells significantly attenuated the anti‐proliferative efficacy of M4, with HSP70 knockdown demonstrating the most significant effect (Figure 4D, Figure S4D). These results imply that HSP70 may serve as a direct molecular target of M4.

Further molecular docking analyses revealed that M4 can bind to the ATP‐binding domain of HSP70 (Figure 4E). This interaction was experimentally confirmed using MST, which demonstrated a direct binding affinity between M4 and HSP70, with a Kd of 26.84 ± 4.91 μM (Figure 4F, Figure S2I). Additionally, DARTS assays provided evidence supporting the direct binding of M4 to HSP70 (Figure 4G). To investigate whether the binding of M4 to HSP70 influences its expression and functional activity, WB analysis was performed using the TNBC cell line MDA‐MB‐231. The results revealed that M4 exerted a minimal effect on the overall expression level of HSP70 (Figure 4H), indicating that M4 likely inhibits HSP70 activity through direct binding rather than by altering its expression levels. Previous studies have revealed that HSP70 regulates its client proteins by interacting with HSP40 via its ATP‐binding domain [46]. To assess whether M4 affects this functional interaction, co‐immunoprecipitation (Co‐IP) assays were conducted to evaluate the binding capacity between HSP70 and HSP40. The findings demonstrated that treatment with M4 significantly reduced the interaction between HSP70 and HSP40 (Figure 4I). These results support the conclusion that M4 exerts anti‐proliferative effects on TNBC cells by binding to HSP70 and subsequently inhibiting its function.

### 3.5 HSP70 Knockdown Abolishes M4‐Mediated Lysosomal Damage and Suppressive Effect on TNBC

**FIGURE 5:**
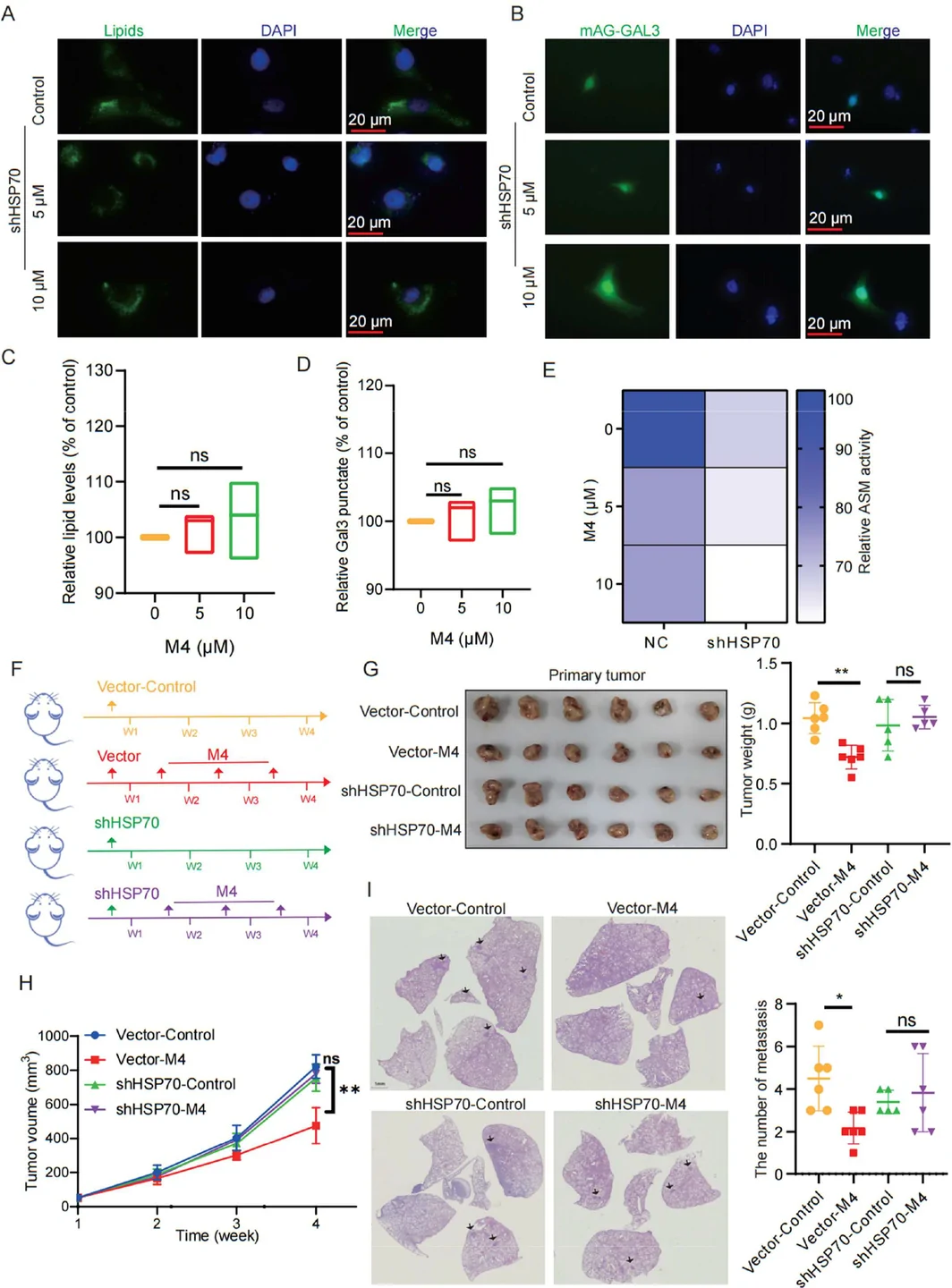
HSP70 knockdown prevents M4‐mediated lysosomal damage and suppression in TNBC. (A) Immunofluorescence analysis of the effects of HSP70 interference on M4‐mediated lipid metabolism. Scale bar: 20 μm. (B) Immunofluorescence analysis of the effect of HSP70 disruption on M4‐mediated lysosomal damage. Scale bar: 20 μm. (C, D) Quantitative diagram of the effects of HSP70 disruption on M4‐mediated lipid metabolism and lysosomal damage. (E) Effects of HSP70 disruption on M4‐mediated ASM activity. (F) Schematic diagram demonstrating that the anti‐TNBC effect of M4 in animal models depends on HSP70. (G) In situ interference with HSP70 inhibited the anti‐TNBC proliferative effect of the M4 compound. The tumour mass was quantified at the experimental endpoint (*n* = 6 mice per group). (H) Tumour volume was quantified throughout the experiment, and tumour mass was measured at the end of the study. (I) H&E staining to measure the anti‐lung metastasis effect of M4 against TNBC following in situ interference with HSP70. Lung metastasis foci were quantified (*n* = 6 mice per group). Data are presented as the mean ± SEM. *p < 0.05, **p < 0.01, ***p < 0.001, ****p < 0.0001 versus the untreated control. ns = no significance. Statistical significance was determined by one‐way ANOVA.

M4 exerts biological activity by binding to and inhibiting HSP70. This prompted us to investigate whether HSP70 knockdown could impair the ability of M4 to disrupt lysosomal function and inhibit proliferation and metastasis in the 4 T1 orthotopic breast cancer model. At the cellular level, HSP70 depletion significantly attenuated M4‐induced lysosomal damage (Figure 5B). Additionally, M4‐mediated lipid accumulation and suppression of ASM activity in MDA‐MB‐231 cells were abolished upon HSP70 knockdown (Figure 5A,C–E, and Figure S4E). A rescue experiment was performed to restore M4 sensitivity in HSP70‐knockdown cells by overexpressing wild‐type HSP70 (Figure S3A,B). These results demonstrate that HSP70 is essential for the disruption of lysosomal integrity and lipid homeostasis by M4.

To evaluate the therapeutic significance in vivo, we administered AAV2‐shHSP70 via intratumoral injection 1 week before M4 treatment. HSP70 knockdown significantly compromised the ability of M4 to suppress tumour growth and pulmonary metastasis (Figure 5F–I, Figure S4F). These findings demonstrate that the antitumor efficacy of M4, including its induction of lysosomal damage, functionally depends on HSP70 inhibition.

### 3.6 Imbalance of HSP70‐Mediated Autophagy Homeostasis Affects the Mesenchymal and Stemness Phenotypes of MDA‐MB‐231 Cell Line

**FIGURE 6:**
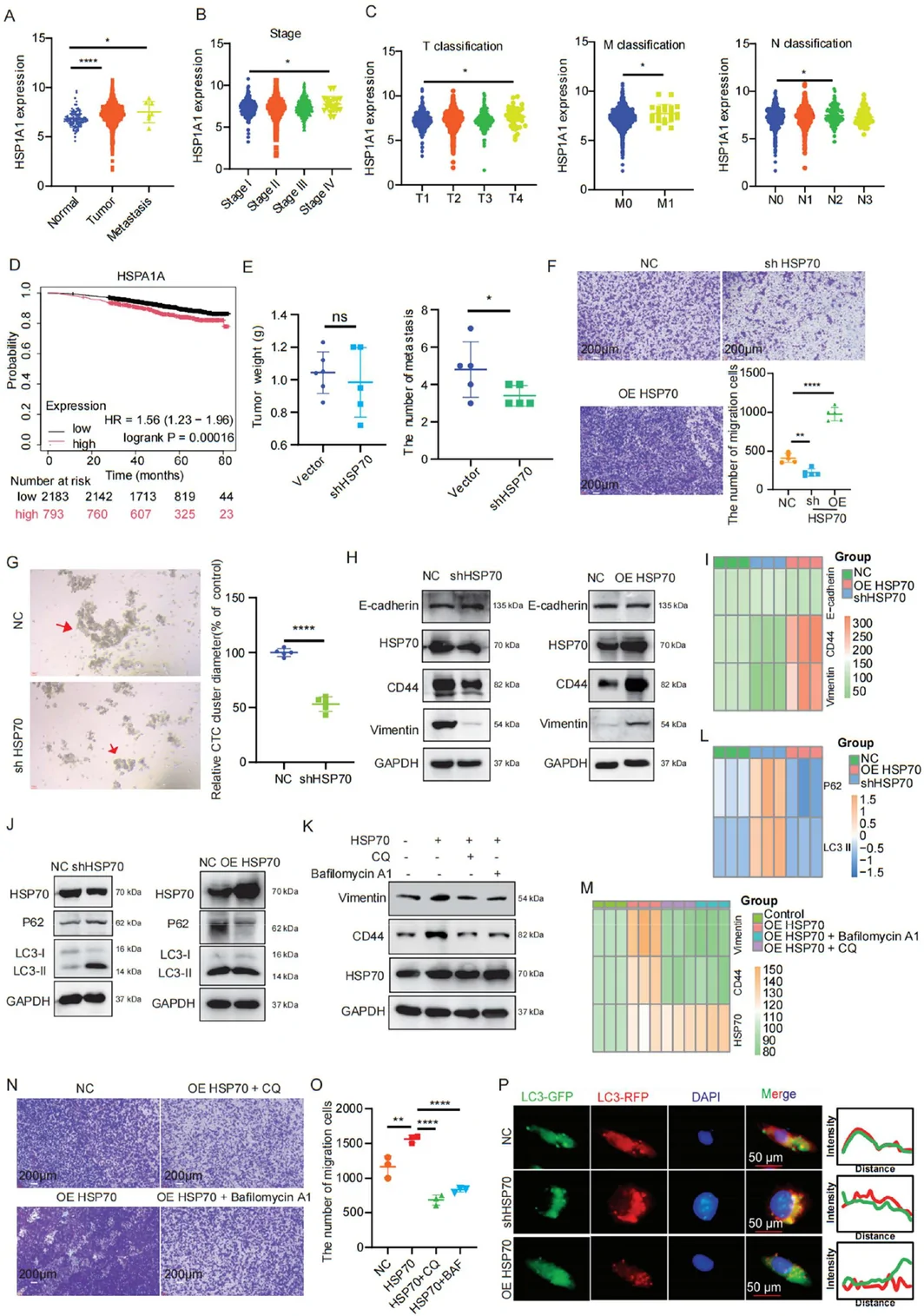
The imbalance of HSP70‐mediated autophagy homeostasis affects mesenchymal and stemness phenotypes of the TNBC cell line. (A) The mRNA expression matrix for 1088 patients with breast cancer was downloaded from the TCGA database, the HSP70 mRNA expression matrix was extracted, and HSP70 mRNA expression was analysed in normal tissue, primary sites, and metastatic sites. (B) Analysis of HSP70 mRNA expression in patients with breast cancer at different stages. (C) The differences in HSP70 mRNA expression among breast cancer patients at different stages were examined according to the clinical TNM staging. (D) Kaplan–Meier overall survival curve analysis: Relationship between HSP70 expression and survival rates in patients with breast cancer. (E) Following HSP70 disruption in 4 T1 in situ breast cancer, changes in tumour mass and lung metastasis were examined (*n* = 6–7 mice per group). Data are presented as the mean ± SEM. (F) Transwell chamber assay was used to investigate the effects of HSP70 disruption and overexpression on the migration capacity of MDA‐MB‐231 cells. Data are presented as the mean ± SD. (G) CTC cluster formation assay was used to investigate the effect of HSP70 inhibition on the diameter of MDA‐MB‐231 CTC clusters. Data are presented as the mean ± SD. (H) Effects of HSP70 disruption and overexpression on the mesenchymal and stemness phenotypes of the MDA‐MB‐231 cell line. (I) Heatmap depicting the quantitative analysis of mesenchymal and stemness‐related proteins in MDA‐MB‐231 cells following HSP70 disruption and overexpression. (J) Effects of HSP70 disruption and overexpression on autophagy‐related proteins in the MDA‐MB‐231 cell line. (K) Effects of autophagy inhibitor pretreatment on HSP70‐mediated mesenchymal and stemness properties in MDA‐MB‐231 cell lines. (L, M) Heatmap illustrating the effects of HSP70 disruption and overexpression on autophagy‐related proteins and proteins associated with mesenchymal and stemness properties in the MDA‐MB‐231 cell line. (N, O) Transwell chamber assays were performed to evaluate the effects of autophagy inhibitor treatment on HSP70 overexpression‐mediated migration capacity in MDA‐MB‐231 cells. Data are presented as the mean ± SD. (P) Effects of HSP70 disruption and overexpression on autophagosomes and autophagolysosomes in the MDA‐MB‐231 cell line. Scale bar: 50 μm. *p < 0.05, **p < 0.01, ***p < 0.001, ****p < 0.0001 versus the untreated control. ns = no significance. Statistical significance was determined by log‐rank test (D), one‐way ANOVA (E, G, N, O), or unpaired two‐tailed Student's *t*‐test (F, where applicable).

HSP70 functional inhibition is crucial for the antitumor activity of M4; however, its specific role in TNBC requires further elucidation. To investigate this, we analysed mRNA expression data and clinical information from 1088 patients with breast cancer in the TCGA database. The analysis revealed significantly elevated HSP70 expression in tumour tissues and metastatic lesions compared with normal adjacent tissues (Figure 6A, Table S3). Furthermore, HSP70 expression gradually increased with advancing clinical stage, a pattern consistently observed across TNM classifications (Figure 6B,C). Survival analysis demonstrated that high HSP70 expression predicted shorter overall survival in both the general breast cancer population and the TNBC subgroup (Figure 6D, Figure S4G). Furthermore, previous studies have demonstrated that intratumoral disruption of HSP70 significantly suppresses lung metastasis while exerting a limited effect on the volume of the primary tumour (Figure 6E). These results establish HSP70 as a key promoter of malignant progression and poor prognosis in breast cancer.

The role of HSP70 in TNBC progression was investigated through an integrated analysis of TCGA data and experimental validation. Transcriptomic analysis of patients with breast cancer stratified by HSP70 expression revealed that high HSP70 levels were associated with enrichment of critical pathways, including hormone metabolism, unfolded protein response, epithelial‐mesenchymal transition (EMT), oxidative phosphorylation, and cell adhesion molecules (Figure S6A, Table S4). Comparative analysis of primary tumours and metastatic lesions revealed that metastases were enriched in wound healing, extracellular matrix organization, and hormone regulation pathways (Figure S4H,I and Table S2). Functional analyses revealed that HSP70 knockdown significantly impaired TNBC cell migration and stem‐like properties in the Transwell and circulating tumour cell cluster formation assays, while HSP70 overexpression increased migratory capacity (Figure 6F,G). Mechanistically, HSP70 positively regulated mesenchymal markers (vimentin and CD44) and suppressed epithelial marker E‐cadherin expression in MDA‐MB‐231 cells (Figure 6H,I, Figure S3C,D). These findings were corroborated by immunohistochemical analysis of orthotopic tumours (Figure S5D,F), confirming HSP70's role in maintaining mesenchymal and stemness phenotypes in TNBC.

Previous research has demonstrated that M4 inhibits autophagic flux in the MDA‐MB‐231 cell line through the involvement of HSP70. Building on this finding, an important question arises: What effect does the modulation of HSP70 expression have on cellular autophagy? WB analysis revealed that HSP70 knockdown led to increased levels of P62 and LC3‐II (Figure 6J,L and Figure S3C,D), two key markers of autophagic flux disruption. Conversely, HSP70 overexpression resulted in lower P62 levels (Figure 6J,L). Furthermore, immunofluorescence studies provided additional evidence that HSP70 knockdown increased the number of autophagosomes within cells and promoted the accumulation of autophagic structures (Figure 6P). These findings imply that functional inhibition of HSP70 impedes the progression of autophagic flux, producing effects similar to those induced by M4. Autophagic homeostasis is defined as the dynamic equilibrium of autophagic flux, in which the formation, transport, and lysosomal degradation of autophagosomes are tightly coordinated to maintain cellular homeostasis. On this basis, HSP70 therefore serves as a critical regulator of autophagic homeostasis.

Based on the established regulatory role of HSP70, we investigated the functional connection between HSP70‐mediated mesenchymal/stemness phenotypes and autophagy in MDA‐MB‐231 cells. Pharmacological inhibition of autophagy using CQ or bafilomycin A1 significantly attenuated the upregulation of vimentin and CD44 induced by HSP70 overexpression (Figure 6K,M and Figure S3E), indicating that autophagy is required for maintaining these EMT and stemness markers. Consistently, Transwell migration assays revealed that autophagy blockade effectively suppressed the enhanced migratory capacity induced by HSP70 overexpression (Figure 6N,Q). These findings indicate that HSP70 regulates EMT and stemness phenotypes in TNBC cells by modulating autophagic flux, highlighting autophagy homeostasis as a critical mechanistic link in HSP70‐mediated tumour progression.

Given that HSP70 interference downregulated EMT and stemness phenotypes in MDA‐MB‐231 cells, we investigated whether the HSP70‐inhibiting compound M4 produces analogous effects. Immunohistochemical analysis of primary tumour tissues revealed that M4 treatment significantly upregulated E‐cadherin while downregulating CD44 expression (Figure S5B,E). Cellular experiments in MDA‐MB‐231 models further confirmed M4's suppression of EMT and stemness markers (Figure S5C). These results demonstrate that pharmacological inhibition of HSP70 by M4 phenocopies the effects of genetic HSP70 interference, establishing HSP70 targeting as the mechanism through which M4 modulates EMT and stemness in TNBC.

### 3.7 M4 Enhances the Antitumor Effects of Paclitaxel Synergistically in TNBC

**FIGURE 7:**
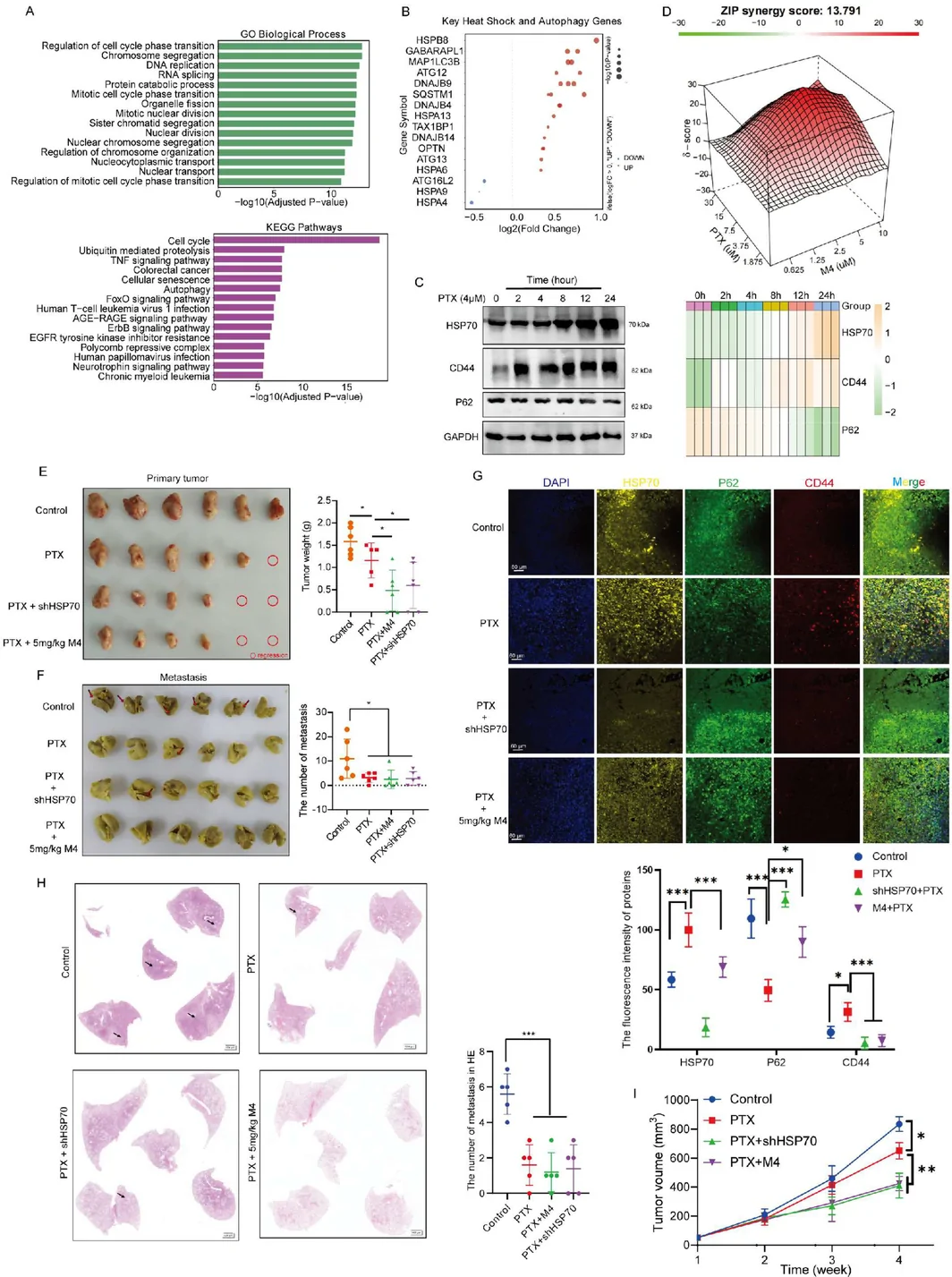
M4 synergistically enhances the antitumor effect of paclitaxel in TNBC. (A) GSE98238 dataset was downloaded from the GEO database. GO and KEGG enrichment analyses were performed on genes with *p* < 0.05. PTX versus Control. (B) The expression profiles of HSPs and autophagy‐related proteins based on logFC. (C) WB assay was performed to detect changes in CD44, P62, and HSP70 expression following PTX treatment. (D) To evaluate the potential synergy of combined drugs, the observed drug combination responses (dose–response matrix or tensor, see figure above) are compared with expected combination responses calculated by means of synergy scoring models (SynergyFinder, https://synergyfinder.fimm.fi). The synergy maps highlight synergistic and antagonistic dose regions in red and green colours, respectively. (E) In vivo animal experiments were performed to investigate the effects of HSP70 disruption and M4 treatment on PTX‐mediated in situ tumour proliferation. The tumour mass was quantified at the experimental endpoint (*n* = 6 mice per group). Data are presented as the mean ± SEM. (F) In vivo animal experiments were performed to investigate the effects of HSP70 disruption and M4 treatment on PTX‐mediated lung metastasis. Lung metastasis was quantified (*n* = 6 mice per group). Data are presented as the mean ± SEM. (G) Multiplex staining was performed on tissue sections of in situ tumours in each group. Scale bar: 60 μm. (H) H&E staining was performed on lung tissue from each group, and the number of metastases in each lung lobe were quantified (*n* = 6 mice per group). Data are presented as the mean ± SEM. (I) Tumour volume was quantified throughout the experiment, and tumour mass was measured at the end of the study. *p < 0.05, **p < 0.01, ***p < 0.001, ****p < 0.0001 versus the untreated control. ns = no significance. Statistical significance was determined by one‐way ANOVA (D, F, H).

Autophagy is widely recognized as a significant contributor to chemotherapy resistance and distant metastasis of tumours [47, 48]. Given this, a pertinent question arises: Can the modulation of the homeostatic function of HSP70‐mediated autophagy improve the efficacy of chemotherapy? In this study, we first obtained the mRNA expression matrix from the GSE98238 dataset, available through the Gene Expression Omnibus (GEO) database (Table S5). This dataset contains mRNA expression profiles of MDA231‐LM2 breast cancer cells before and after paclitaxel treatment. Subsequently, gene ontology (GO) and Kyoto encyclopedia of genes and genomes (KEGG) pathway analyses were conducted on this data. The results revealed that Paclitaxel treatment suppressed the cell cycle and DNA replication in breast cancer cells (Figure 7A). However, it concurrently induced several processes, including cellular senescence, cytokine secretion, and autophagy. These findings demonstrate that autophagy is one of the key stress response mechanisms activated in breast cancer cells following paclitaxel treatment. Furthermore, the expression patterns of autophagy‐related proteins and HSPs were determined. Paclitaxel treatment upregulated the mRNA levels of autophagy‐associated genes and members of the HSP70 family (Figure 7B, Figure S6B). At the cellular level, we confirmed that paclitaxel treatment caused a time‐dependent increase in HSP70 and CD44 expression, as well as a decrease in P62 expression (Figure 7C). These cellular changes support the conclusion that paclitaxel treatment activates autophagy, thereby leading to elevated HSP70 expression.

Furthermore, we hypothesized that disrupting HSP70 function or treating cells with M4 could enhance the proliferative inhibition induced by paclitaxel in the MDA‐MB‐231 cell line, while simultaneously suppressing its migratory capacity. Colony formation assays revealed that both HSP70 disruption and M4 treatment significantly increased the ability of paclitaxel to inhibit the proliferation of MDA‐MB‐231 cells (Figure S5G). Conversely, HSP70 overexpression failed to produce such an enhancing effect (Figure S5G). Additionally, we investigated the effect of low‐dose paclitaxel on the migration of MDA‐MB‐231 cells. These findings demonstrated that exposure to low‐dose paclitaxel promoted the migratory ability of these cells (Figure S3G,I). However, this pro‐migratory effect was effectively counteracted when HSP70 was disrupted or when cells were treated with the M4 compound. These results imply that targeting HSP70 not only augments the anti‐proliferative efficacy of paclitaxel but also attenuates the migration‐promoting effect induced by low‐dose paclitaxel.

M4 enhances the antitumor effects of paclitaxel synergistically in TNBC cells (Figure S3G,I). Furthermore, in vivo experiments were conducted to evaluate the effect of HSP70 targeting on the anti‐breast cancer efficacy of paclitaxel. The findings demonstrated that paclitaxel treatment effectively inhibited the growth of 4 T1 orthotopic breast tumours in terms of volume and mass and significantly suppressed lung metastasis, with one mouse exhibiting complete tumour regression. Treatment of mice with the M4 compound or intratumoral interference targeting HSP70 resulted in a reduction in the volume and mass of the orthotopic tumours, along with enhanced inhibition of lung metastasis. In these groups, two mice in each cohort demonstrated regression of the orthotopic tumour (Figure 7D,F,H, Figure S5H). These results demonstrate that targeting HSP70 can improve the anti‐breast cancer effects of paclitaxel. Additionally, multiplex immunohistochemical analysis of the orthotopic breast tumours revealed that paclitaxel treatment increased the expression of HSP70 and CD44, while concurrently decreasing P62 expression in the tumours (Figure 7G). Conversely, HSP70 disruption and M4 treatment alleviated the paclitaxel‐induced changes, providing evidence that targeting HSP70 synergistically enhances the antitumor activity of paclitaxel. These results demonstrate that targeting HSP70 can synergize with paclitaxel to exert anti‐TNBC effects.

## 4 Discussion

**FIGURE 8:**
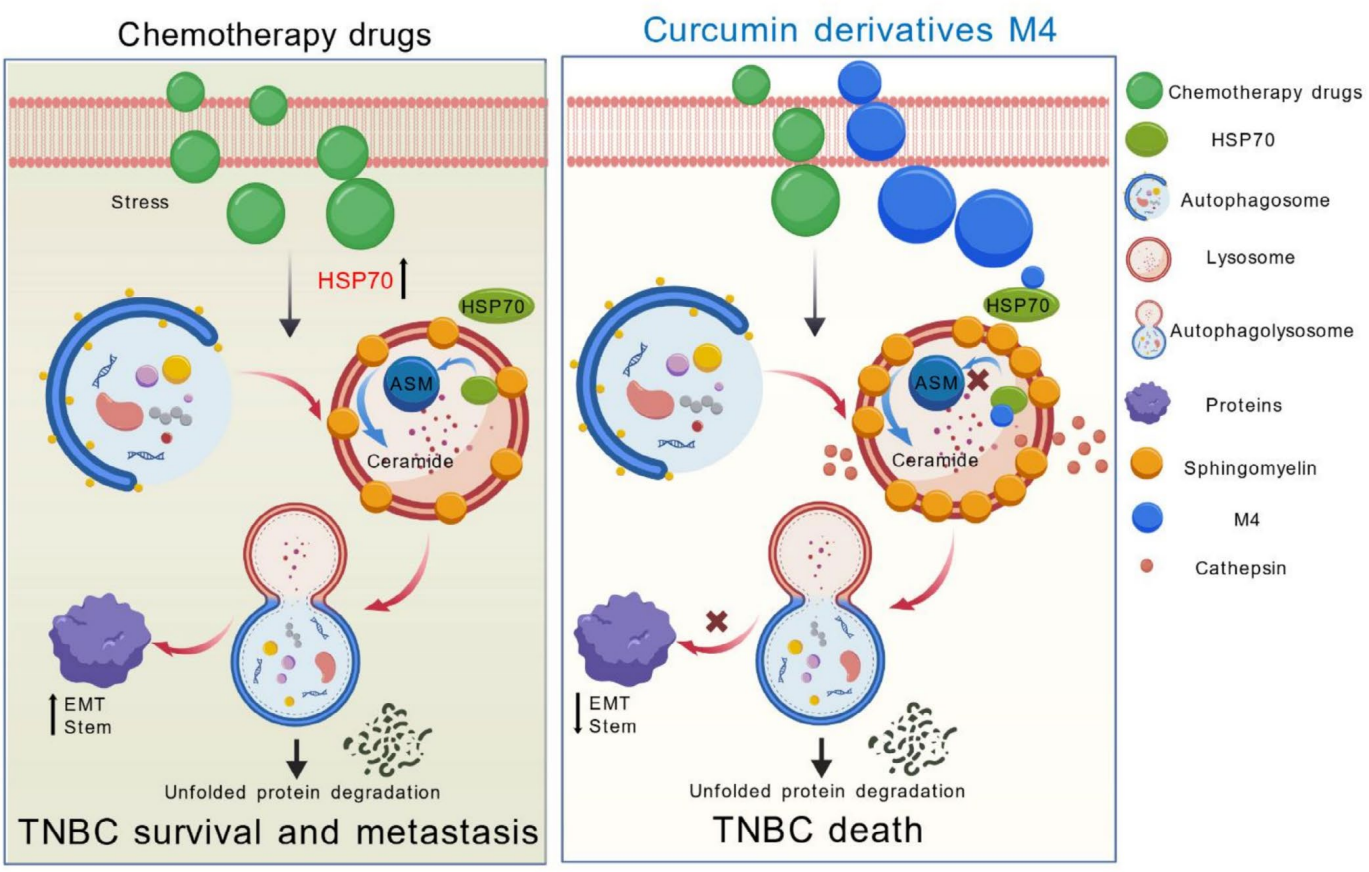
Mechanism of this study. Chemotherapy drug treatment increased HSP70 expression, maintaining autophagy homeostasis to mediate EMT in TNBC cell lines, ultimately promoting cell survival and metastasis. Co‐treatment with compound M4 blocked this process, primarily by binding to HSP70 and disrupting its function (image drawn at https://biogdp.com/).

A novel curcumin derivative, M4, was identified in this study, exhibiting potent anti‐TNBC activity. Mechanistically, M4, a weakly basic compound, neutralizes lysosomal acidity and increases lysosomal membrane permeability. This disruption leads to autophagic flux blockade and apoptosis in TNBC cells. Furthermore, we identified HSP70 as a molecular target of M4. M4 binds to the ATP‐binding domain of HSP70, thereby inhibiting its activity and contributing to its strong anti‐breast cancer activity. M4 induces lysosomal alkalization and, by targeting HSP70, triggers lysosomal cathepsin release to activate apoptotic signalling, thereby exerting an anti‐tumour effect. Additionally, our results revealed that HSP70 functions as a key regulatory gene involved in TNBC metastasis. HSP70 modulates the mesenchymal phenotype and the stemness characteristics of TNBC cell lines by maintaining the homeostasis of autophagic processes. Moreover, our findings revealed that targeting HSP70 in combination with the chemotherapeutic agent paclitaxel produces a synergistic anti‐cancer effect in TNBC (Figure 8).

Developing curcumin derivatives is a vital strategy to overcome the pharmacokinetic limitations of natural curcumin, including poor solubility and low bioavailability, while enhancing its anti‐cancer efficacy [49, 50, 51]. These structurally modified compounds not only improve target specificity and metabolic stability but also hold promise for the discovery of novel mechanisms of action against breast cancer. Our recent identification of curcumin derivative M4 demonstrates this potential; while its primary target is HSP70, evidence suggests additional unidentified targets, particularly in inhibiting metastasis. Taken together, our integrative strategy consisting of DARTS‐MS dataset, TCGA‐based TNBC patient analysis, and RVS‐predicted targets enabled systematic identification of M4‐binding candidates. Among the eight overlapping proteins initially identified from DARTS‐MS and CPTAC datasets, several including CD44, EGFR, SRC, and TRIM25 have been functionally linked to autophagy and lysosomal dynamics, representing putative secondary or associated targets worthy of further investigation. None of these candidates, however, were consistently supported by all three orthogonal approaches. In contrast, HSP70 was the only protein identified across all three datasets, and was further validated by functional studies showing that genetic inhibition of HSP70 markedly blunted the anti‐proliferative activity of M4. These findings confirm HSP70 as a bona fide direct target of M4, whereas other early‐screening candidates remain unvalidated putative secondary interactors. Multiple pieces of evidence support HSP70 as a key target; DARTS assays indicate the existence of other potential binding proteins. This is consistent with the behaviour of many small‐molecule drugs, whose biological effects may arise from multi‐target interactions. Although incompletely characterized, this multi‐target engagement profile highlights the advantages of curcumin derivatives over traditional single‐target therapies. The ongoing research on these novel compounds is expanding the therapeutic arsenal and improving our understanding of breast cancer biology. By optimizing the structure of curcumin, more effective agents can be developed capable of simultaneously targeting multiple hallmarks of cancer, including proliferation, apoptosis resistance, and metastasis. This approach underscores the transition from the parent compound to more advanced derivatives as a promising pathway for future cancer therapeutics.

Chemotherapy remains a cornerstone in the treatment of TNBC; however, it has significant limitations, including the enhancement of autophagy and cancer stem cell characteristics that contribute to recurrence, therapy resistance, and metastasis [7, 47, 52, 53]. Targeting autophagy has emerged as a promising combinatorial strategy. For example, the combination of CQ, an autophagy inhibitor, with doxorubicin has been demonstrated to improve therapeutic efficacy and decrease tumour viability in TNBC models [9, 54, 55]. Additionally, combining drugs such as metformin with chemotherapy has demonstrated improved outcomes due to improved autophagic modulation [49, 56, 57]. New, more effective autophagy inhibitors should be developed in the future that can be used alongside chemotherapy to improve therapeutic outcomes in TNBC. By optimizing these combinations, we may improve the clinical efficiency and patient outcomes for this aggressive cancer subtype.

HSP70 plays essential roles in cellular stress responses, protein homeostasis, and apoptosis regulation, which are pivotal in TNBC progression [58, 59]. Targeting HSP70 has demonstrated significant therapeutic potential; for example, inhibiting HSP70 sensitizes TNBC cells to doxorubicin [60, 61]. Besides, HSP70 is involved in autophagy regulation, thereby influencing cancer cell survival under stress. HSP70 facilitates autophagic processes, promoting TNBC cell survival during chemotherapy [62, 63]. In this study, chemotherapy increased HSP70 levels, which can contribute to resistance against therapy. Consequently, combining HSP70 inhibitors with chemotherapeutics may enhance treatment efficacy. Our results demonstrated that M4 effectively targets HSP70, inhibiting its function and disrupting autophagic flux, thereby enhancing the efficacy of paclitaxel in TNBC treatment. This indicates that targeting HSP70 is a promising adjunctive strategy for chemotherapy in TNBC.

In summary, we identified a novel curcumin derivative, M4, exhibiting potent antitumor activity and synergistically increasing the effects of paclitaxel in TNBC. Moreover, this study highlights that targeting HSP70 is a new therapeutic strategy for combination treatments in TNBC management. These findings underscore the potential of HSP70 as a significant target for improving the efficacy of existing chemotherapy regimens. By integrating M4 with HSP70 inhibition into treatment protocols, we may expand therapeutic options for patients with TNBC, paving the way for more effective clinical treatments. Due to limitations associated with the current research stage and available resources, this study has not yet performed a systematic pharmacokinetic and toxicological evaluation of M4. Future studies should assess the plasma half‐life, tissue distribution in major organs, maximum tolerated dose, and preliminary safety profiles of M4 in mouse or large animal models. Such data will lay a solid foundation for the further preclinical development of M4.

## Author Contributions

**Zijian Li:** data curation, formal analysis, investigation, funding acquisition, writing – review and editing. **Wanxia Wang:** investigation, validation, formal analysis, methodology. **Yuxin Zhou:** data curation, formal analysis, writing – review and editing. **Tao Zeng:** visualization. **Jian Liu:** conceptualisation, data curation, resources, supervision, writing – original draft. **Yingjie Qing:** data curation, resources, supervision, formal analysis, methodology. **Xuan Han:** supervision, project administration, funding acquisition, writing – review and editing.

## Funding

This work was supported by the Excellent Innovation Talent Cultivation Project, 035062005004‐56, the National Natural Science Foundation of China, 82204696.

## Conflicts of Interest

The authors declare no conflicts of interest.

## Supporting information


**Table S1:** for Darts, RVS, TNBC vs. non‐TNBC data.

**Table S2:** for comparative analysis of metastatic vs. primary.

**Table S3:** for HSPA1A expression in breast cancer patients from the TCGA database.

**Table S4:** for bioinformatics analysis HSPA1A expression (high vs. low) in breast cancer patients from the TCGA database.

**Table S5:** for bioinformatics analysis HSPA1A expression (high vs. low) from GSE98238.

**Figure S1:** (A) 1 H NMR spectrum of compound N17. (B) 13 C NMR spectrum of compound N17. (C) Structure of curcumin derivative M5. (D) Synthesis pathways of the N series of curcumin derivatives. (E) MTT assay to evaluate the effects of curcumin derivative M5 and N‐series compounds on cell proliferation in breast cancer cell lines. The N‐series served as positive controls. (F) MTT assay to evaluate the proliferation‐inhibitory effect of curcumin derivative compound M4 on MCF‐10A cells. (G) WB assay to investigate the effects of curcumin derivative compound M4 on apoptosis‐related proteins in the 4 T1 cell line. (H) Clonogenic assay was used to investigate the effect of autophagy inhibitors on M4‐mediated proliferation inhibition. 4 T1 or MDA‐MB‐231 cells were pretreated with the indicated inhibitors for 1 h, followed by co‐treatment with various concentrations of M4 for 24 h. The inhibitors and their final concentrations were: Chloroquine (CQ, 20 μM), Bafilomycin A1 (Baf A1, 100 nM), MG‐132 (10 μM), 3‐Methyladenine (3‐MA, 5 mM), and a GSK3β inhibitor (10 μM). Cell viability was assessed by colony formation assay. The results were analysed quantitatively. (I) Quantitative diagram depicting the effects of autophagy inhibitors on the M4‐mediated expression of autophagy‐related proteins. (J) H&E staining was used to investigate the effects of M4 on liver and kidney damage. Bar, SD. *p < 0.05, **p < 0.01, ***p < 0.001, ****p < 0.0001 versus the untreated control.
**Figure S2:** (A) WB assay for evaluating the effects of compound M4 on apoptosis‐related proteins in the MCF‐7 cell line. (B) Effects of M4 compound treatment on autophagy‐related proteins LC3B and P62 in the 4 T1 cell line. (C) WB analysis investigating the effects of pretreatment with autophagy inhibitors CQ and bafilomycin on autophagy‐related proteins mediated by the M4 compound. (D) Effects of M4 compound treatment on LAMP1 in the MDA‐MB‐231 cell line. (E) The lysosomal probe was labelled in the MCF‐7 cell line, and flow cytometry was used to investigate the effect of M4 on lysosomal acidity. (F) Enrichment analysis of differentially expressed proteins between TNBC and non‐TNBC tumours was performed using data extracted from the CPTAC breast cancer cohort. (G) An integrated multi‐omics screening strategy was used to systematically identify the potential protein targets of compound M4 in TNBC (Created with Bohrium). (H) Phosphate standard curve for the ATPase assay. The absorbance at 630 nm (A630) was plotted against phosphate standards (nmol), showing a linear correlation (*R*^2 = 0.9944). Relative ATPase activity of HSP70 treated with indicated concentrations of M4. VER‐15508 (25 uM) was used as a positive inhibitor control. Data represent mean ± SD (*n* = 3). *p < 0.05, ***p < 0.001 vs. control; NS, not significant. (I) MST was used to perform a competitive binding experiment by co‐treating HSP70 with VER‐155008 and M4. (J) WB analysis can be used to perform the expression of HSP70 in different cell lines. TNBC cell lines (1: MDA‐MB‐468, 2: MDA‐MB‐231, 3: 4 T1) and non‐TNBC cell lines (4: T‐47D, 5: MCF‐7).
**Figure S3:** (A) Rescue experiments were used to examine the role of HSP70 in M4‐mediated apoptosis by overexpressing HSP70 in HSP70 knockdown cell lines. (B) Effects of HSP70 disruption on M4‐mediated ASM activity. (C‐D) Effects of HSP70 disruption and overexpression on the mesenchymal, stemness phenotypes and autophagy‐related proteins of the 4 T1 cell line. (E) Effects of autophagy inhibitor pretreatment on HSP70‐mediated mesenchymal and stemness properties in 4 T1 cell lines. (F) The lysosomal probe was labelled in the MDA‐MB‐231 and HSP70 disruption MDA‐MB‐231 cell line, and flow cytometry was used to investigate the effect of M4 on lysosomal acidity. (G) Transwell chamber assay was used to evaluate the effects of HSP70 inhibition and M4 treatment on PTX‐mediated migration capacity. (H) MTT assay showing the effect of different concentrations of paclitaxel on cell viability in MDA‐MB‐231 cell line. (I) Wound‐healing assay was used to evaluate the effects of HSP70 inhibition and M4 treatment on PTX‐mediated migration capacity. (J) Effect of combined treatment with different concentrations of M4 and paclitaxel on the viability of MDA‐MB‐231 cell line.
**Figure S4:** (A) DARTS experiment was performed to investigate protein changes following treatment with compound M4. (B) Cellular distribution of differentially expressed proteins following treatment with M4. (C) Reactome enrichment analysis of differentially expressed proteins following treatment with M4. (D) MTT assay was used to investigate the effects of HSP70 and HSP90 inhibition on the proliferation‐inhibitory activity of M4 in MDA‐MB‐231 cells. (E) Investigating the effects of HSP70 and HSP90 disruption on the M4‐mediated inhibition of ASM activity in MDA‐MB‐231 cell lines. (F) WB analysis validating HSP70 disruption. (G) Kaplan–Meier overall survival curve analysis: The relationship between HSP70 expression and survival rates in patients with TNBC. (H) mRNA expression matrices for patients with in situ and metastatic breast cancer were downloaded from TCGA. GO and KEGG enrichment analyses were performed on genes with *p* < 0.05. (I) Comparison of changes in autophagy, EMT, and stemness‐related pathway genes between patients with in situ breast cancer and metastatic breast cancer. Bar, SD. *p < 0.05, **p < 0.01, ***p < 0.001, ****p < 0.0001 versus the untreated control.
**Figure S5:** (A) Immunohistochemical analysis to investigate the effects of M4 and PTX treatment on the expression of cleaved caspase‐3, CTSD, and LC3B in the in situ tumour. The expression of each protein was quantified. Scale bar: 100 μm. (B) Immunohistochemical analysis evaluating the effects of M4 on E‐cadherin and CD44 expression in in situ tumours. Scale bar: 100 μm. (C) WB assay investigating the effects of M4 on the expression of E‐cadherin, vimentin, and CD44 in the MDA‐MB‐231 cell line. (D) Immunohistochemical analysis revealed that HSP70 disrupted the expression of E‐cadherin, p62, and CD44 in the in situ tumour model. Scale bar: 100 μm. (E) Quantitative analysis of the effects of M4 on E‐cadherin and CD44 expression in the in situ tumours. (F) Quantitative analysis of the effects of HSP70 disruption on the expression of E‐cadherin, P62, and CD44 in orthotopic tumours. (G) Effects of HSP70 and M4 disruption or overexpression on PTX‐mediated proliferation capacity in MDA‐MB‐231 cell lines. Quantification of cell proliferation capacity. (H) Effect of HSP70 disruption and M4 processing on PTX‐mediated antitumor effects. Statistical analysis of mouse body volume throughout the experiment. Bar, SD. *p < 0.05, **p < 0.01, ***p < 0.001, ****p < 0.0001 versus the untreated control.
**Figure S6:** (A) Breast cancer patients' data were downloaded from TCGA and grouped based on the average mRNA expression of HSPA1A. Genes with *p* < 0.05 underwent GO and KEGG enrichment analyses. High HSP1A1 versus low HSP1A1. (B) Visualization of HSPs and autophagy‐related proteins in the GSE98238 dataset using heatmap analysis. Comparison between the paclitaxel and control groups. Bar, SD. *p < 0.05, **p < 0.01, ***p < 0.001, ****p < 0.0001 versus the untreated control.

## Data Availability

The data that support the findings of this study are available from the corresponding author upon reasonable request.
