# Supplementary material for: A Curcumin‐Derived HSP70 Inhibitor Disrupts Lysosomal Function to Suppress Triple‐Negative Breast Cancer Progression
Source: Cell Prolif. 2026 Apr 29;59(9):e70222. doi: 10.1111/cpr.70222 (PMC13325827; doi:10.1111/cpr.70222)
Supplement: Supplementary file 1 [file CPR-59-e70222-s002.docx]

**Supplementary Figure S1.** (**A**) 1 H NMR spectrum of compound N17. (**B**) 13 C NMR spectrum of compound N17. (**C**) Structure of curcumin derivative M5. (**D**) Synthesis pathways of the N series of curcumin derivatives. (**E**) MTT assay to evaluate the effects of curcumin derivative M5 and N-series compounds on cell proliferation in breast cancer cell lines. The N-series served as positive controls. (**F**) MTT assay to evaluate the proliferation-inhibitory effect of curcumin derivative compound M4 on MCF-10A cells. (**G**) WB assay to investigate the effects of curcumin derivative compound M4 on apoptosis-related proteins in the 4T1 cell line. (**H**) Clonogenic assay was used to investigate the effect of autophagy inhibitors on M4-mediated proliferation inhibition. 4T1 or MDA-MB-231 cells were pretreated with the indicated inhibitors for 1 hour, followed by co-treatment with various concentrations of M4 for 24 hours. The inhibitors and their final concentrations were: Chloroquine (CQ, 20 μM), Bafilomycin A1 (Baf A1, 100 nM), MG-132 (10 μM), 3-Methyladenine (3-MA, 5 mM), and a GSK3β inhibitor (10 μM). Cell viability was assessed by colony formation assay. The results were analyzed quantitatively. (**I**) Quantitative diagram depicting the effects of autophagy inhibitors on the M4-mediated expression of autophagy-related proteins. (**J**) H&E staining was used to investigate the effects of M4 on liver and kidney damage. Bar, SD. **P* < 0.05, ***P* < 0.01, ****P* < 0.001, *****P* < 0.0001 versus the untreated control.

**Supplementary Figure S2.** (**A**) WB assay for evaluating the effects of compound M4 on apoptosis-related proteins in the MCF-7 cell line. (**B**) Effects of M4 compound treatment on autophagy-related proteins LC3B and P62 in the 4T1 cell line. (**C**) WB analysis investigating the effects of pretreatment with autophagy inhibitors CQ and bafilomycin on autophagy-related proteins mediated by the M4 compound. (**D**) Effects of M4 compound treatment on LAMP1 in the MDA-MB-231 cell line. (**E**) The lysosomal probe was labeled in the MCF-7 cell line, and flow cytometry was used to investigate the effect of M4 on lysosomal acidity. (**F**) Enrichment analysis of differentially expressed proteins between TNBC and non-TNBC tumors was performed using data extracted from the CPTAC breast cancer cohort. (**G**) An integrated multi-omics screening strategy was used to systematically identify the potential protein targets of compound M4 in TNBC (Created with Bohrium). (**H**) Phosphate standard curve for the ATPase assay. The absorbance at 630 nm (A630) was plotted against phosphate standards (nmol), showing a linear correlation (R^2 = 0.9944). Relative ATPase activity of HSP70 treated with indicated concentrations of M4. VER-15508 (25 uM) was used as a positive inhibitor control. Data represent mean ± SD (n=3). *P < 0.05, ***P < 0.001 vs. control; NS, not significant. (**I**) MST was used to perform a competitive binding experiment by co-treating HSP70 with VER-155008 and M4. (**J**) WB analysis can be used to perform the expression of HSP70 in different cell lines. TNBC cell lines (1: MDA-MB-468, 2: MDA-MB-231, 3: 4T1) and non-TNBC cell lines (4: T-47D, 5: MCF-7).

**Supplementary Figure S3.** (**A**) Rescue experiments were used to examine the role of HSP70 in M4-mediated apoptosis by overexpressing HSP70 in HSP70 knockdown cell lines. (**B**) Effects of HSP70 disruption on M4-mediated ASM activity. (**C-D**) Effects of HSP70 disruption and overexpression on the mesenchymal, stemness phenotypes and autophagy-related proteins of the 4T1 cell line. (**E**) Effects of autophagy inhibitor pretreatment on HSP70-mediated mesenchymal and stemness properties in 4T1 cell lines. (**F**) The lysosomal probe was labeled in the MDA-MB-231 and HSP70 disruption MDA-MB-231 cell line, and flow cytometry was used to investigate the effect of M4 on lysosomal acidity. (**G**) Transwell chamber assay was used to evaluate the effects of HSP70 inhibition and M4 treatment on PTX-mediated migration capacity. (**H**) MTT assay showing the effect of different concentrations of paclitaxel on cell viability in MDA-MB-231 cell line. (**I**) Wound-healing assay was used to evaluate the effects of HSP70 inhibition and M4 treatment on PTX-mediated migration capacity. (**J**) Effect of combined treatment with different concentrations of M4 and paclitaxel on the viability of MDA-MB-231 cell line.

**Supplementary Figure S4.** (**A**) DARTS experiment was performed to investigate protein changes following treatment with compound M4. (**B**) Cellular distribution of differentially expressed proteins following treatment with M4. (**C**) Reactome enrichment analysis of differentially expressed proteins following treatment with M4. (**D**) MTT assay was used to investigate the effects of HSP70 and HSP90 inhibition on the proliferation-inhibitory activity of M4 in MDA-MB-231 cells. (**E**) Investigating the effects of HSP70 and HSP90 disruption on the M4-mediated inhibition of ASM activity in MDA-MB-231 cell lines. (**F**) WB analysis validating HSP70 disruption. (**G**) Kaplan–Meier overall survival curve analysis: The relationship between HSP70 expression and survival rates in patients with TNBC. (**H**) mRNA expression matrices for patients with *in situ* and metastatic breast cancer were downloaded from TCGA. GO and KEGG enrichment analyses were performed on genes with *P* < 0.05. (**I**) Comparison of changes in autophagy, EMT, and stemness-related pathway genes between patients with *in situ* breast cancer and metastatic breast cancer. Bar, SD. **P* < 0.05, ***P* < 0.01, ****P* < 0.001, *****P* < 0.0001 versus the untreated control.

**Supplementary Figure S5.** (**A**) Immunohistochemical analysis to investigate the effects of M4 and PTX treatment on the expression of cleaved caspase-3, CTSD, and LC3B in the *in situ* tumor. The expression of each protein was quantified. Scale bar: 100 μm. (**B**) Immunohistochemical analysis evaluating the effects of M4 on E-cadherin and CD44 expression in *in situ* tumors. Scale bar: 100 μm. (**C**) WB assay investigating the effects of M4 on the expression of E-cadherin, vimentin, and CD44 in the MDA-MB-231 cell line. (**D**) Immunohistochemical analysis revealed that HSP70 disrupted the expression of E-cadherin, p62, and CD44 in the *in situ* tumor model. Scale bar: 100 μm. (**E**) Quantitative analysis of the effects of M4 on E-cadherin and CD44 expression in the *in situ* tumors. (**F**) Quantitative analysis of the effects of HSP70 disruption on the expression of E-cadherin, P62, and CD44 in orthotopic tumors. (**G**) Effects of HSP70 and M4 disruption or overexpression on PTX-mediated proliferation capacity in MDA-MB-231 cell lines. Quantification of cell proliferation capacity. (**H**) Effect of HSP70 disruption and M4 processing on PTX-mediated antitumor effects. Statistical analysis of mouse body volume throughout the experiment. Bar, SD. **P* < 0.05, ***P* < 0.01, ****P* < 0.001, *****P* < 0.0001 versus the untreated control.

**Supplementary Figure S6.** (**A**) Breast cancer patients' data were downloaded from TCGA and grouped based on the average mRNA expression of HSPA1A. Genes with *P* < 0.05 underwent GO and KEGG enrichment analyses. High HSP1A1 versus low HSP1A1. (**B**) Visualization of HSPs and autophagy-related proteins in the GSE98238 dataset using heatmap analysis. Comparison between the paclitaxel and control groups. Bar, SD. **P* < 0.05, ***P* < 0.01, ****P* < 0.001, *****P* < 0.0001 versus the untreated control.
