# Supplementary material for: A Curcumin‐Derived HSP70 Inhibitor Disrupts Lysosomal Function to Suppress Triple‐Negative Breast Cancer Progression
Source: Cell Prolif. 2026 Apr 29:e70222. Online ahead of print. doi: 10.1111/cpr.70222 (PMC13325827; doi:10.1111/cpr.70222)
Supplement: Supplementary file 6 — Figure S1: (A) 1 H NMR spectrum of compound N17. (B) 13 C NMR spectrum of compound N17. (C) Structure of curcumin derivative M5. (D) Synthesis pathways of the N series of curcumin derivatives. (E) MTT assay to evaluate the effects of curcumin derivative M5 and N‐series compounds on cell proliferation in breast cancer cell lines. The N‐series served as positive controls. (F) MTT assay to evaluate the proliferation‐inhibitory effect of curcumin derivative compound M4 on MCF‐10A cells. (G) WB assay to investigate the effects of curcumin derivative compound M4 on apoptosis‐related proteins in the 4 T1 cell line. (H) Clonogenic assay was used to investigate the effect of autophagy inhibitors on M4‐mediated proliferation inhibition. 4 T1 or MDA‐MB‐231 cells were pretreated with the indicated inhibitors for 1 h, followed by co‐treatment with various concentrations of M4 for 24 h. The inhibitors and their final concentrations were: Chloroquine (CQ, 20 μM), Bafilomycin A1 (Baf A1, 100 nM), MG‐132 (10 μM), 3‐Methyladenine (3‐MA, 5 mM), and a GSK3β inhibitor (10 μM). Cell viability was assessed by colony formation assay. The results were analysed quantitatively. (I) Quantitative diagram depicting the effects of autophagy inhibitors on the M4‐mediated expression of autophagy‐related proteins. (J) H&E staining was used to investigate the effects of M4 on liver and kidney damage. Bar, SD. *p < 0.05, **p < 0.01, ***p < 0.001, ****p < 0.0001 versus the untreated control. Figure S2: (A) WB assay for evaluating the effects of compound M4 on apoptosis‐related proteins in the MCF‐7 cell line. (B) Effects of M4 compound treatment on autophagy‐related proteins LC3B and P62 in the 4 T1 cell line. (C) WB analysis investigating the effects of pretreatment with autophagy inhibitors CQ and bafilomycin on autophagy‐related proteins mediated by the M4 compound. (D) Effects of M4 compound treatment on LAMP1 in the MDA‐MB‐231 cell line. (E) The lysosomal probe was labelled in the MCF‐7 cell line, [file CPR-9999-e70222-s006.docx]

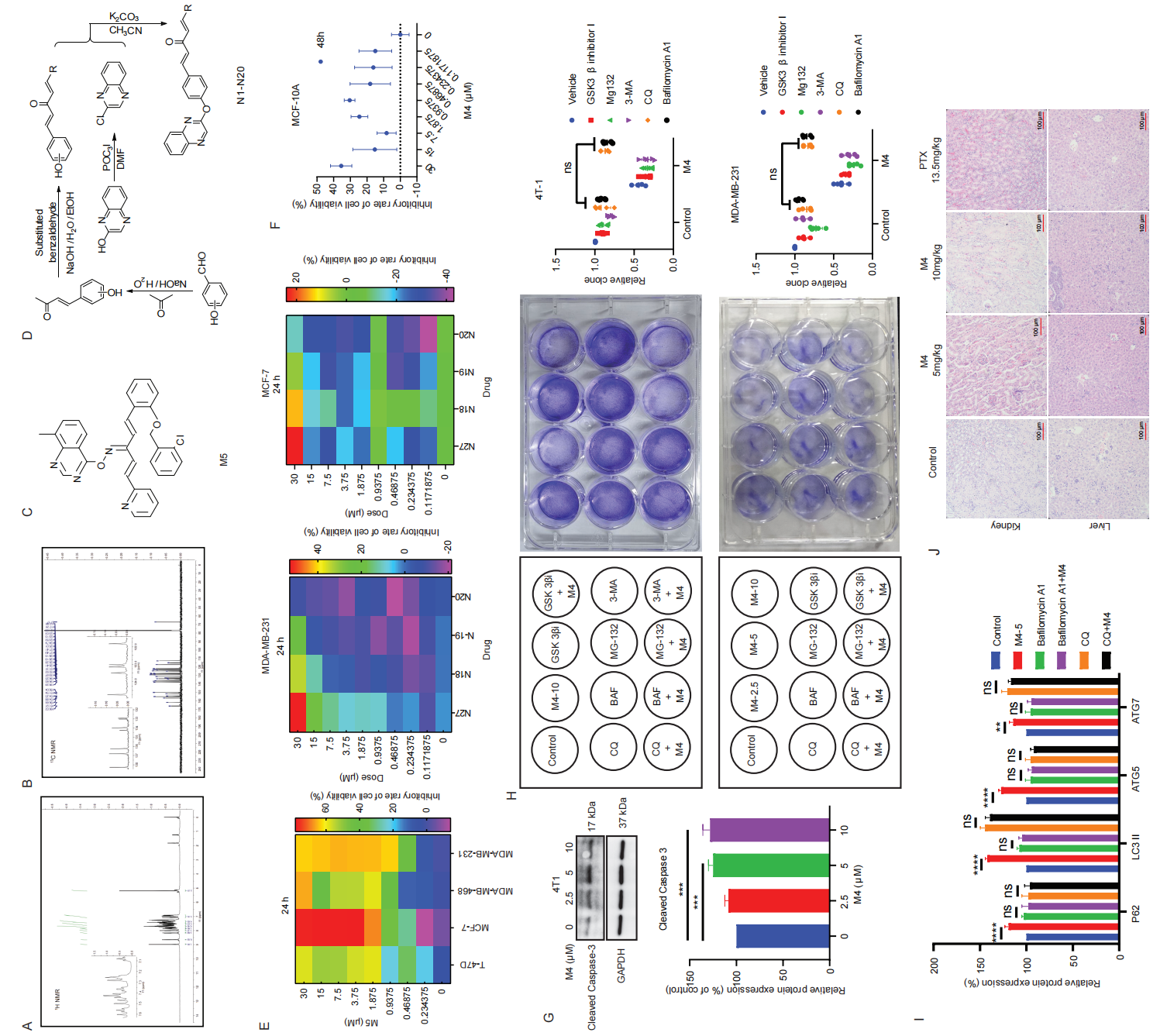


**Supplementary Figure S1.** (**A**) 1 H NMR spectrum of compound N17. (**B**) 13 C NMR spectrum of compound N17. (**C**) Structure of curcumin derivative M5. (**D**) Synthesis pathways of the N series of curcumin derivatives. (**E**) MTT assay to evaluate the effects of curcumin derivative M5 and N-series compounds on cell proliferation in breast cancer cell lines. The N-series served as positive controls. (**F**) MTT assay to evaluate the proliferation-inhibitory effect of curcumin derivative compound M4 on MCF-10A cells. (**G**) WB assay to investigate the effects of curcumin derivative compound M4 on apoptosis-related proteins in the 4T1 cell line. (**H**) Clonogenic assay was used to investigate the effect of autophagy inhibitors on M4-mediated proliferation inhibition. 4T1 or MDA-MB-231 cells were pretreated with the indicated inhibitors for 1 hour, followed by co-treatment with various concentrations of M4 for 24 hours. The inhibitors and their final concentrations were: Chloroquine (CQ, 20 μM), Bafilomycin A1 (Baf A1, 100 nM), MG-132 (10 μM), 3-Methyladenine (3-MA, 5 mM), and a GSK3β inhibitor (10 μM). Cell viability was assessed by colony formation assay. The results were analyzed quantitatively. (**I**) Quantitative diagram depicting the effects of autophagy inhibitors on the M4-mediated expression of autophagy-related proteins. (**J**) H&E staining was used to investigate the effects of M4 on liver and kidney damage. Bar, SD. **P* < 0.05, ***P* < 0.01, ****P* < 0.001, *****P* < 0.0001 versus the untreated control.


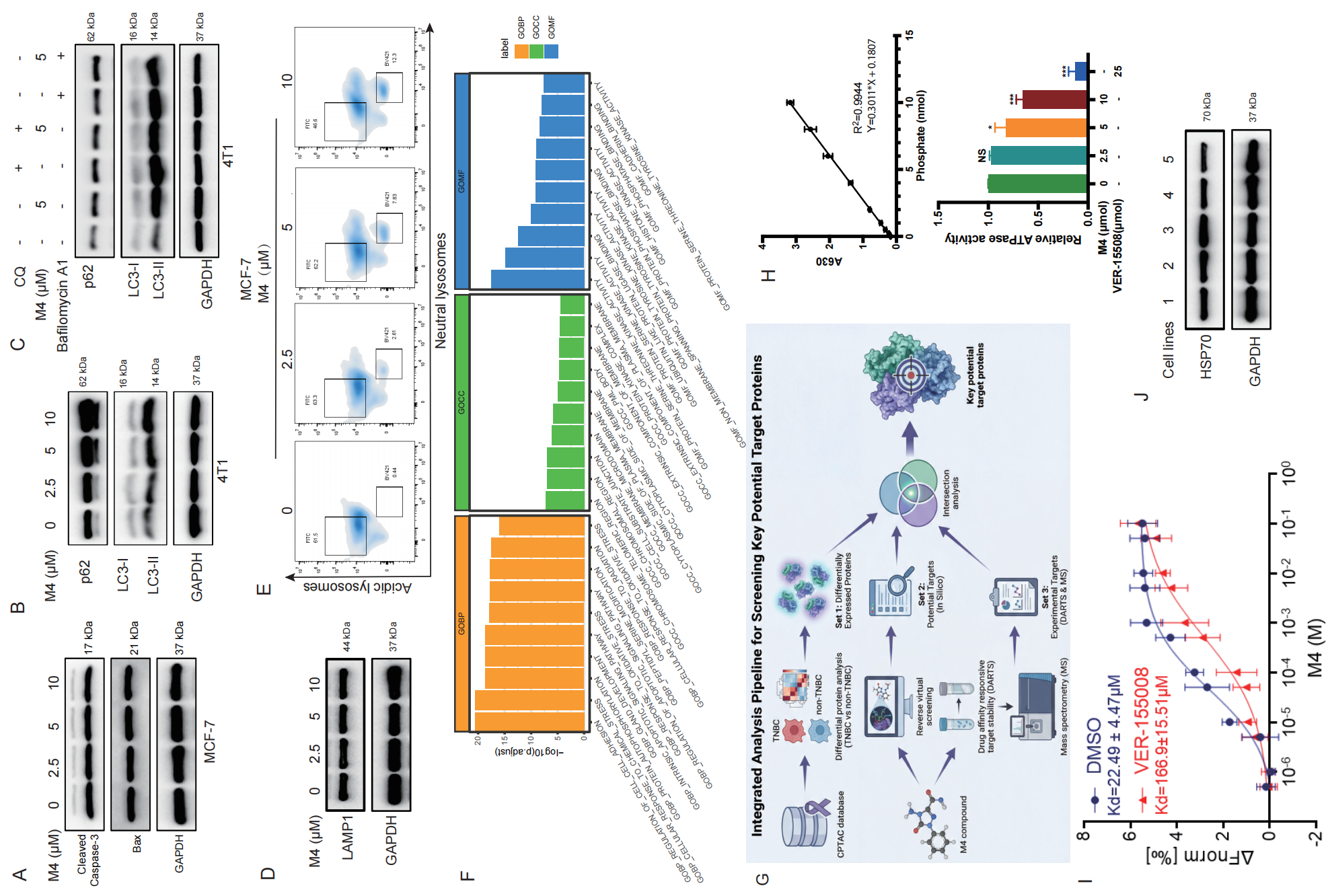


**Supplementary Figure S2.** (**A**) WB assay for evaluating the effects of compound M4 on apoptosis-related proteins in the MCF-7 cell line. (**B**) Effects of M4 compound treatment on autophagy-related proteins LC3B and P62 in the 4T1 cell line. (**C**) WB analysis investigating the effects of pretreatment with autophagy inhibitors CQ and bafilomycin on autophagy-related proteins mediated by the M4 compound. (**D**) Effects of M4 compound treatment on LAMP1 in the MDA-MB-231 cell line. (**E**) The lysosomal probe was labeled in the MCF-7 cell line, and flow cytometry was used to investigate the effect of M4 on lysosomal acidity. (**F**) Enrichment analysis of differentially expressed proteins between TNBC and non-TNBC tumors was performed using data extracted from the CPTAC breast cancer cohort. (**G**) An integrated multi-omics screening strategy was used to systematically identify the potential protein targets of compound M4 in TNBC (Created with Bohrium). (**H**) Phosphate standard curve for the ATPase assay. The absorbance at 630 nm (A630) was plotted against phosphate standards (nmol), showing a linear correlation (R^2 = 0.9944). Relative ATPase activity of HSP70 treated with indicated concentrations of M4. VER-15508 (25 uM) was used as a positive inhibitor control. Data represent mean ± SD (n=3). *P < 0.05, ***P < 0.001 vs. control; NS, not significant. (**I**) MST was used to perform a competitive binding experiment by co-treating HSP70 with VER-155008 and M4. (**J**) WB analysis can be used to perform the expression of HSP70 in different cell lines. TNBC cell lines (1: MDA-MB-468, 2: MDA-MB-231, 3: 4T1) and non-TNBC cell lines (4: T-47D, 5: MCF-7).


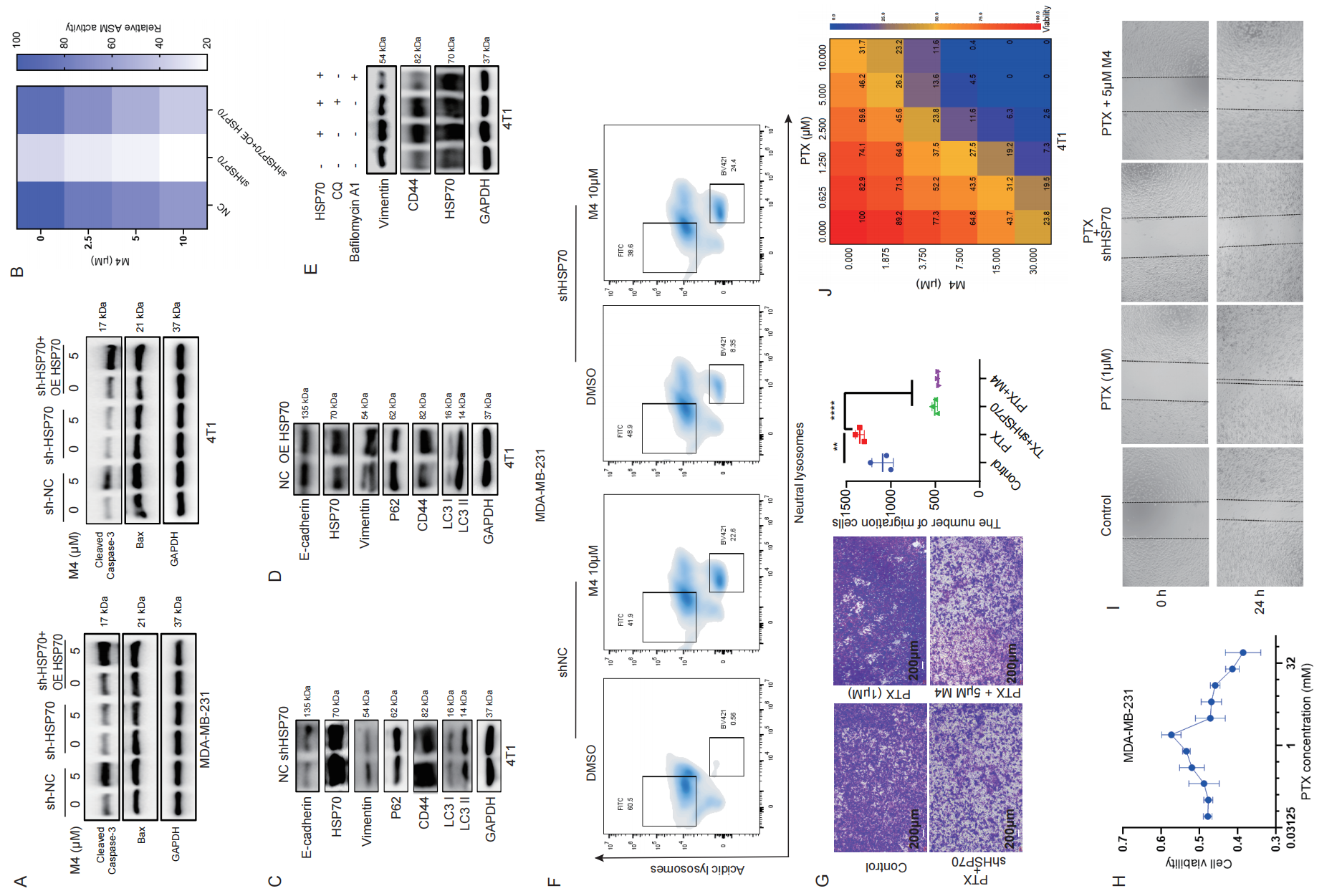


**Supplementary Figure S3.** (**A**) Rescue experiments were used to examine the role of HSP70 in M4-mediated apoptosis by overexpressing HSP70 in HSP70 knockdown cell lines. (**B**) Effects of HSP70 disruption on M4-mediated ASM activity. (**C-D**) Effects of HSP70 disruption and overexpression on the mesenchymal, stemness phenotypes and autophagy-related proteins of the 4T1 cell line. (**E**) Effects of autophagy inhibitor pretreatment on HSP70-mediated mesenchymal and stemness properties in 4T1 cell lines. (**F**) The lysosomal probe was labeled in the MDA-MB-231 and HSP70 disruption MDA-MB-231 cell line, and flow cytometry was used to investigate the effect of M4 on lysosomal acidity. (**G**) Transwell chamber assay was used to evaluate the effects of HSP70 inhibition and M4 treatment on PTX-mediated migration capacity. (**H**) MTT assay showing the effect of different concentrations of paclitaxel on cell viability in MDA-MB-231 cell line. (**I**) Wound-healing assay was used to evaluate the effects of HSP70 inhibition and M4 treatment on PTX-mediated migration capacity. (**J**) Effect of combined treatment with different concentrations of M4 and paclitaxel on the viability of MDA-MB-231 cell line.


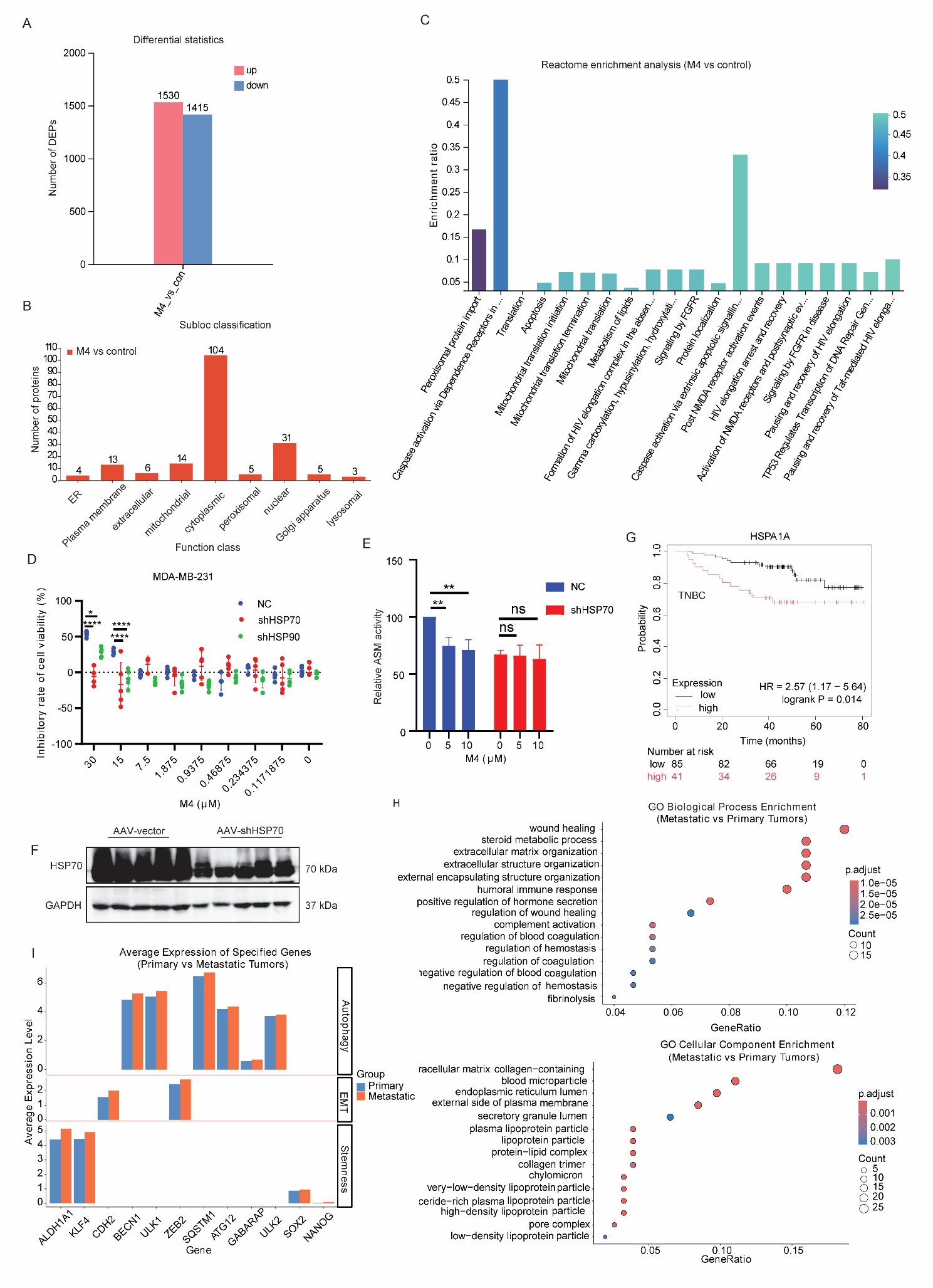


**Supplementary Figure S4.** (**A**) DARTS experiment was performed to investigate protein changes following treatment with compound M4. (**B**) Cellular distribution of differentially expressed proteins following treatment with M4. (**C**) Reactome enrichment analysis of differentially expressed proteins following treatment with M4. (**D**) MTT assay was used to investigate the effects of HSP70 and HSP90 inhibition on the proliferation-inhibitory activity of M4 in MDA-MB-231 cells. (**E**) Investigating the effects of HSP70 and HSP90 disruption on the M4-mediated inhibition of ASM activity in MDA-MB-231 cell lines. (**F**) WB analysis validating HSP70 disruption. (**G**) Kaplan–Meier overall survival curve analysis: The relationship between HSP70 expression and survival rates in patients with TNBC. (**H**) mRNA expression matrices for patients with *in situ* and metastatic breast cancer were downloaded from TCGA. GO and KEGG enrichment analyses were performed on genes with *P* < 0.05. (**I**) Comparison of changes in autophagy, EMT, and stemness-related pathway genes between patients with *in situ* breast cancer and metastatic breast cancer. Bar, SD. **P* < 0.05, ***P* < 0.01, ****P* < 0.001, *****P* < 0.0001 versus the untreated control.


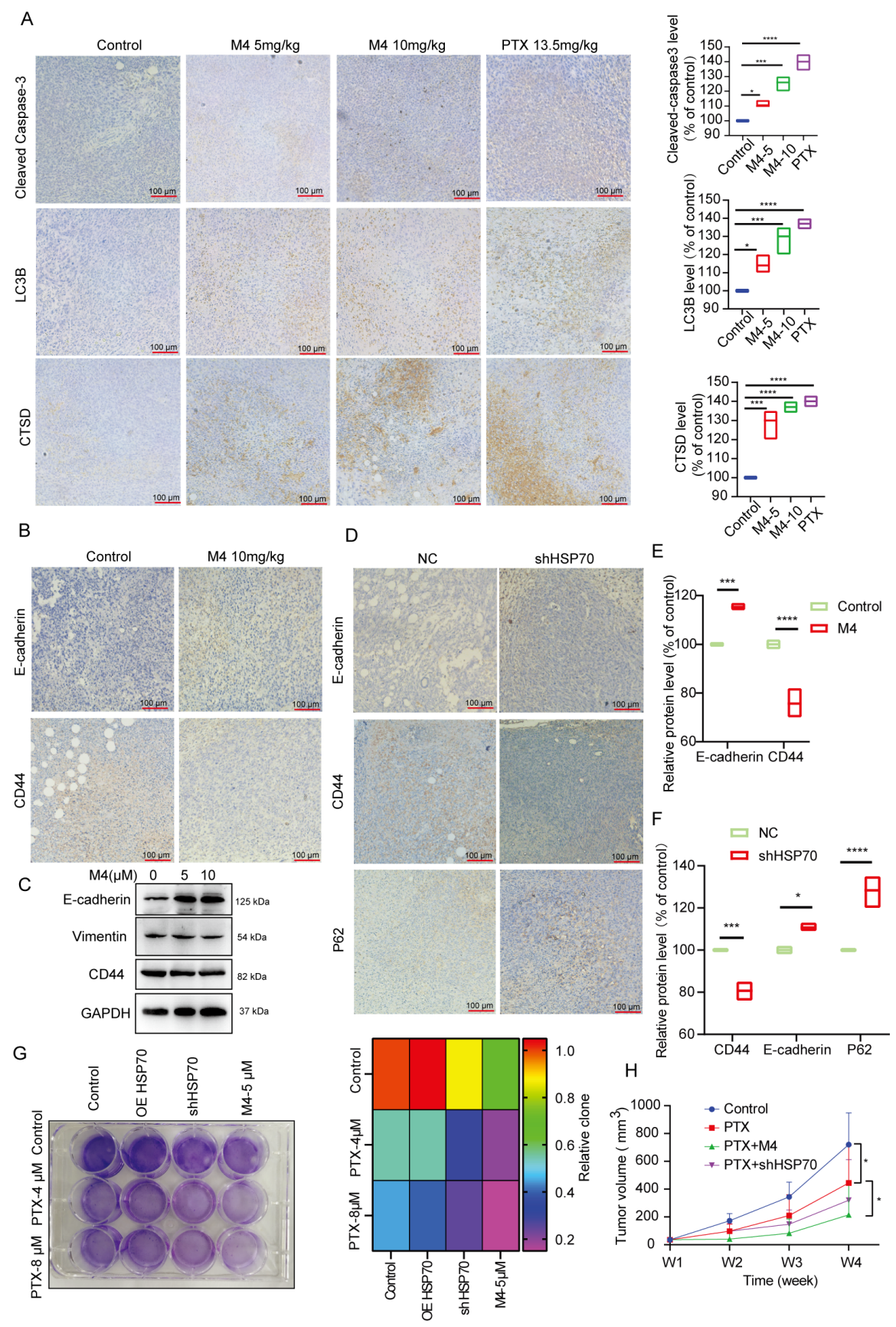


**Supplementary Figure S5.** (**A**) Immunohistochemical analysis to investigate the effects of M4 and PTX treatment on the expression of cleaved caspase-3, CTSD, and LC3B in the *in situ* tumor. The expression of each protein was quantified. Scale bar: 100 μm. (**B**) Immunohistochemical analysis evaluating the effects of M4 on E-cadherin and CD44 expression in *in situ* tumors. Scale bar: 100 μm. (**C**) WB assay investigating the effects of M4 on the expression of E-cadherin, vimentin, and CD44 in the MDA-MB-231 cell line. (**D**) Immunohistochemical analysis revealed that HSP70 disrupted the expression of E-cadherin, p62, and CD44 in the *in situ* tumor model. Scale bar: 100 μm. (**E**) Quantitative analysis of the effects of M4 on E-cadherin and CD44 expression in the *in situ* tumors. (**F**) Quantitative analysis of the effects of HSP70 disruption on the expression of E-cadherin, P62, and CD44 in orthotopic tumors. (**G**) Effects of HSP70 and M4 disruption or overexpression on PTX-mediated proliferation capacity in MDA-MB-231 cell lines. Quantification of cell proliferation capacity. (**H**) Effect of HSP70 disruption and M4 processing on PTX-mediated antitumor effects. Statistical analysis of mouse body volume throughout the experiment. Bar, SD. **P* < 0.05, ***P* < 0.01, ****P* < 0.001, *****P* < 0.0001 versus the untreated control.


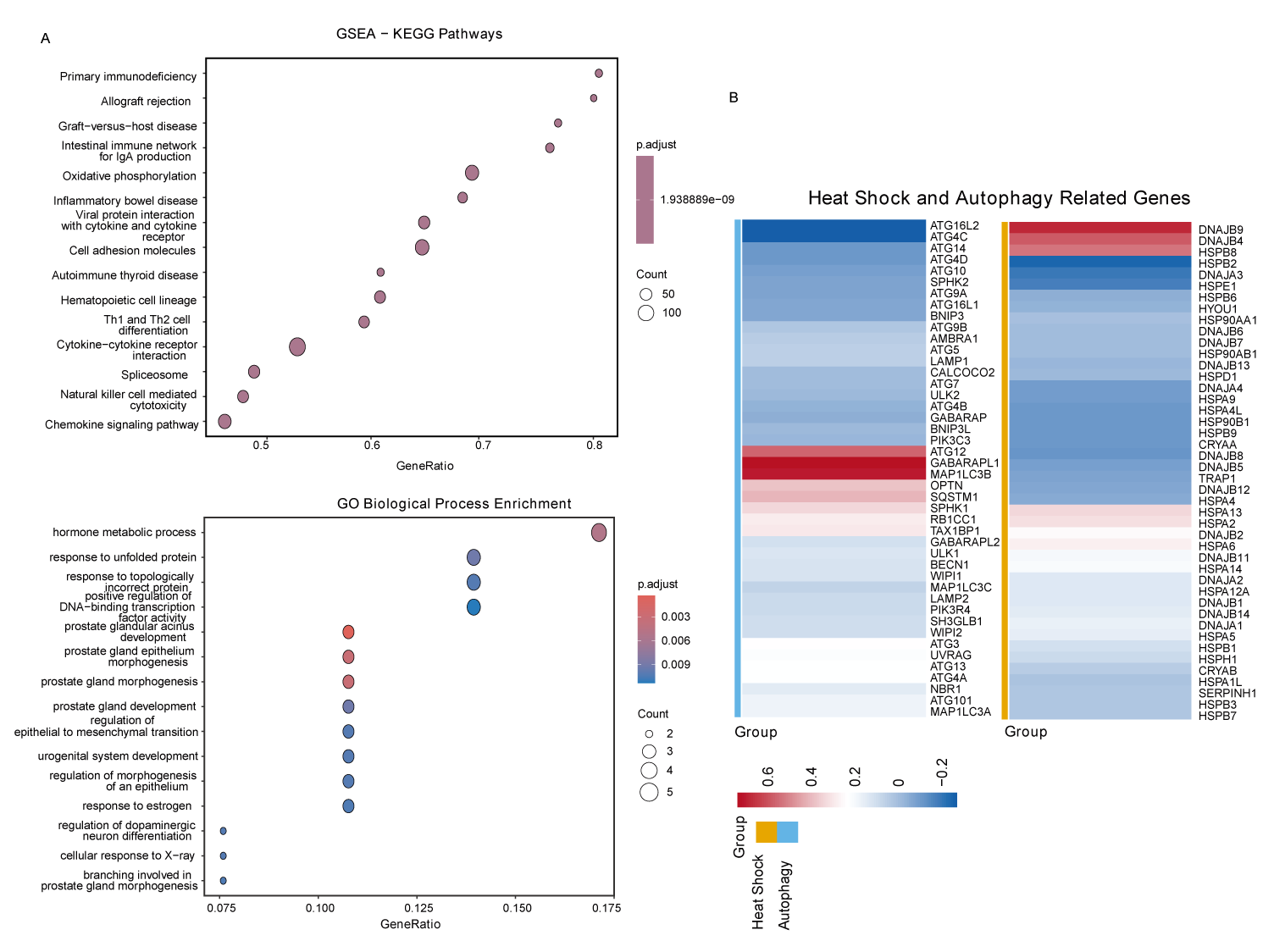


**Supplementary Figure S6.** (**A**) Breast cancer patients' data were downloaded from TCGA and grouped based on the average mRNA expression of HSPA1A. Genes with *P* < 0.05 underwent GO and KEGG enrichment analyses. High HSP1A1 versus low HSP1A1. (**B**) Visualization of HSPs and autophagy-related proteins in the GSE98238 dataset using heatmap analysis. Comparison between the paclitaxel and control groups. Bar, SD. **P* < 0.05, ***P* < 0.01, ****P* < 0.001, *****P* < 0.0001 versus the untreated control.
